# Genetic differentiation and phylogeography of partially sympatric species complex *Rhizophora mucronata* Lam. and *R. stylosa* Griff. using SSR markers

**DOI:** 10.1186/s12862-015-0331-3

**Published:** 2015-03-29

**Authors:** Alison K S Wee, Koji Takayama, Jasher L Chua, Takeshi Asakawa, Sankararamasubramanian H Meenakshisundaram, Onrizal, Bayu Adjie, Erwin Riyanto Ardli, Sarawood Sungkaew, Norhaslinda Binti Malekal, Nguyen Xuan Tung, Severino G Salmo, Orlex Baylen Yllano, M Nazre Saleh, Khin Khin Soe, Yoichi Tateishi, Yasuyuki Watano, Shigeyuki Baba, Edward L Webb, Tadashi Kajita

**Affiliations:** Department of Biology, Graduate School of Science, Chiba University, 1-33 Yayoi-cho, Inage-ku, Chiba, 263-8522 Japan; Present Address: Center for Integrative Conservation, Xishuangbanna Tropical Botanical Garden, Chinese Academy of Sciences, Menglun, Mengla, Yunnan, 666303 China; The University Museum, The University of Tokyo, Hongo 7-3-1, Tokyo, 113-0033 Japan; Department of Biological Sciences, National University of Singapore, Singapore, 117543 Singapore; Biotechnology Programme, M.S. Swaminathan Research Foundation, Chennai, India; Forestry Sciences Department, Universitas Sumatera Utara, Medan, Indonesia; Bali Botanical Garden, Indonesian Institute of Sciences, Bali, Indonesia; Faculty of Biology, Jenderal Soedirman University, Purwokerto, Indonesia; Forest Biology Department, Faculty of Forestry, Kasetsart University, Bangkok, Thailand; Institute for Tropical Biology and Conservation, Universiti Malaysia Kota Kinabalu, Kota Kinabalu, Malaysia; Mangrove Ecosystem Research Centre, Hanoi National University of Education, Hanoi, Vietnam; Department of Environmental Science, School of Science and Engineering, Ateneo de Manila University, Quezon City, Philippines; Biology Department, College of Science and Technology, Adventist University of the Philippines, Silang, 4118 Cavite, Philippines; Department of Forest Production, Faculty of Forestry, Universiti Putra Malaysia, Serdang, Malaysia; Department of Botany, University of Yangon, Yangon, Myanmar; Faculty of Education, University of the Ryukyus, Senbaru, Okinawa, Japan; Tropical Biosphere Research Center, University of the Ryukyus, Iriomote, Okinawa, Japan; International Society for Mangrove Ecosystems (ISME), c/o Faculty of Agriculture, University of the Ryukyus, Senbaru, Okinawa, 903-0129 Japan

**Keywords:** Biogeography, Coastal plants, Gene flow, Genetic diversity, Genetic structure, Indo-West Pacific, Mangrove, Microsatellite, Nuclear DNA, Population genetics

## Abstract

**Background:**

Mangrove forests are ecologically important but globally threatened intertidal plant communities. Effective mangrove conservation requires the determination of species identity, management units, and genetic structure. Here, we investigate the genetic distinctiveness and genetic structure of an iconic but yet taxonomically confusing species complex *Rhizophora mucronata* and *R. stylosa* across their distributional range, by employing a suite of 20 informative nuclear SSR markers.

**Results:**

Our results demonstrated the general genetic distinctiveness of *R. mucronata* and *R. stylosa*, and potential hybridization or introgression between them. We investigated the population genetics of each species without the putative hybrids, and found strong genetic structure between oceanic regions in both *R. mucronata* and *R. stylosa*. In *R. mucronata*, a strong divergence was detected between populations from the Indian Ocean region (Indian Ocean and Andaman Sea) and the Pacific Ocean region (Malacca Strait, South China Sea and Northwest Pacific Ocean). In *R. stylosa*, the genetic break was located more eastward, between populations from South and East China Sea and populations from the Southwest Pacific Ocean. The location of these genetic breaks coincided with the boundaries of oceanic currents, thus suggesting that oceanic circulation patterns might have acted as a cryptic barrier to gene flow.

**Conclusions:**

Our findings have important implications on the conservation of mangroves, especially relating to replanting efforts and the definition of evolutionary significant units in *Rhizophora* species. We outlined the genetic structure and identified geographical areas that require further investigations for both *R. mucronata* and *R. stylosa*. These results serve as the foundation for the conservation genetics of *R. mucronata* and *R. stylosa* and highlighted the need to recognize the genetic distinctiveness of closely-related species, determine their respective genetic structure, and avoid artificially promoting hybridization in mangrove restoration programmes.

**Electronic supplementary material:**

The online version of this article (doi:10.1186/s12862-015-0331-3) contains supplementary material, which is available to authorized users.

## Background

Mangrove forests are ecologically important but globally threatened intertidal plant communities [1]. Despite their crucial roles as sediment filter [2], carbon sink [3], breeding ground for coastal fauna [4] and coastal defense against storm surges [5] and tsunami [6], mangroves are facing global habitat loss—mainly due to land conversion—that surpasses those for other terrestrial ecosystems [7]. Increased awareness of mangrove loss has led to a surge in mangrove conservation worldwide, especially following the deadly tsunami in 2004 [8]. Effective mangrove conservation depends upon contemporary knowledge on taxonomy and phylogeography to clarify species identity, define management units, identify genetic structure and understand population connectivity [9,10]. In the long term, these should serve to preserve the evolutionary potential of mangroves via the identification and subsequent conservation of evolutionary significant units (ESUs). In this regard, genetic studies have contributed substantially by resolving taxonomical uncertainties [11,12] and identifying genetic stocks [13,14].

One of the most pressing species identity issues in mangroves concerns the iconic genus *Rhizophora*. In the Indo-West-Pacific (IWP), *Rhizophora* consists of three endemic species (*R. apiculata*, *R. mucronata* and *R. stylosa*), a variant of *R. mangle* from the Atlantic-East Pacific (AEP) that colonized the IWP (*R. samoensis*) [12], and two hybrids *R.* × *annamalayana* (*R. apiculata* × *R. mucronata*) and *R.* × *lamarckii* (*R. apiculata* × *R. stylosa*) [15-18]. *Rhizophora* is the most popular genus for mangrove restoration in the IWP [19-21], yet the distinction between the two major IWP *Rhizophora* species *R. mucronata* and *R. stylosa*, has remained elusive. Whereas *Rhizophora apiculata* is morphologically [15,22] and genetically [12,23] distinct from *R. mucronata* and *R. stylosa*, the latter two species are morphologically and genetically similar, and were even suggested to be variants of the same species [22]. *Rhizophora mucronata* and *R. stylosa* are dominant in the west and east IWP, respectively [24,25], with overlaps in distribution in Southeast Asia, Northwest Pacific Ocean and northern Australia (Duke et al. [22]). The diagnostic characteristics of these two species—the leaf morphology and the length of the style and propagule—were observed to have substantial intra-species variation and inter-species overlaps [18,22,26]. These possibly lead to local taxonomic confusion. For example, both *R. mucronata* and *R. stylosa* were reported to be dominant in Japan, even though only one common morphotype was observed [24,27,28]. The wide distribution range, large variation in morphological diagnostic characters, and the putative occurrence of hybridization thus undermines a clear distinction between *R. mucronata* and *R. stylosa*, presenting practical challenges to effective conservation.

Recent molecular studies are yet to resolve the taxonomic confusion between the two species. Phylogenetic analyses across the IWP with chloroplast DNA (cpDNA) and nuclear ITS sequence data did not support monophyly for either species, suggesting that they are genetically proximate and/or have experienced gene flow via introgressive hybridization [24,29]. This is further supported by evidence of a proposed natural hybrid between *R. mucronata* and *R. stylosa* in Malaysia [18]. Nevertheless, population genetics based on nuclear inter simple sequence repeat (ISSR) data [24] and nuclear genes [18] were able to discriminate the two species in sympatric populations, suggesting a certain level of reproductive isolation and genetic distinctiveness between them. However, whether the genetic distinctiveness can be found across the distributional range of the sibling species remain to be confirmed.

**Figure 1:**
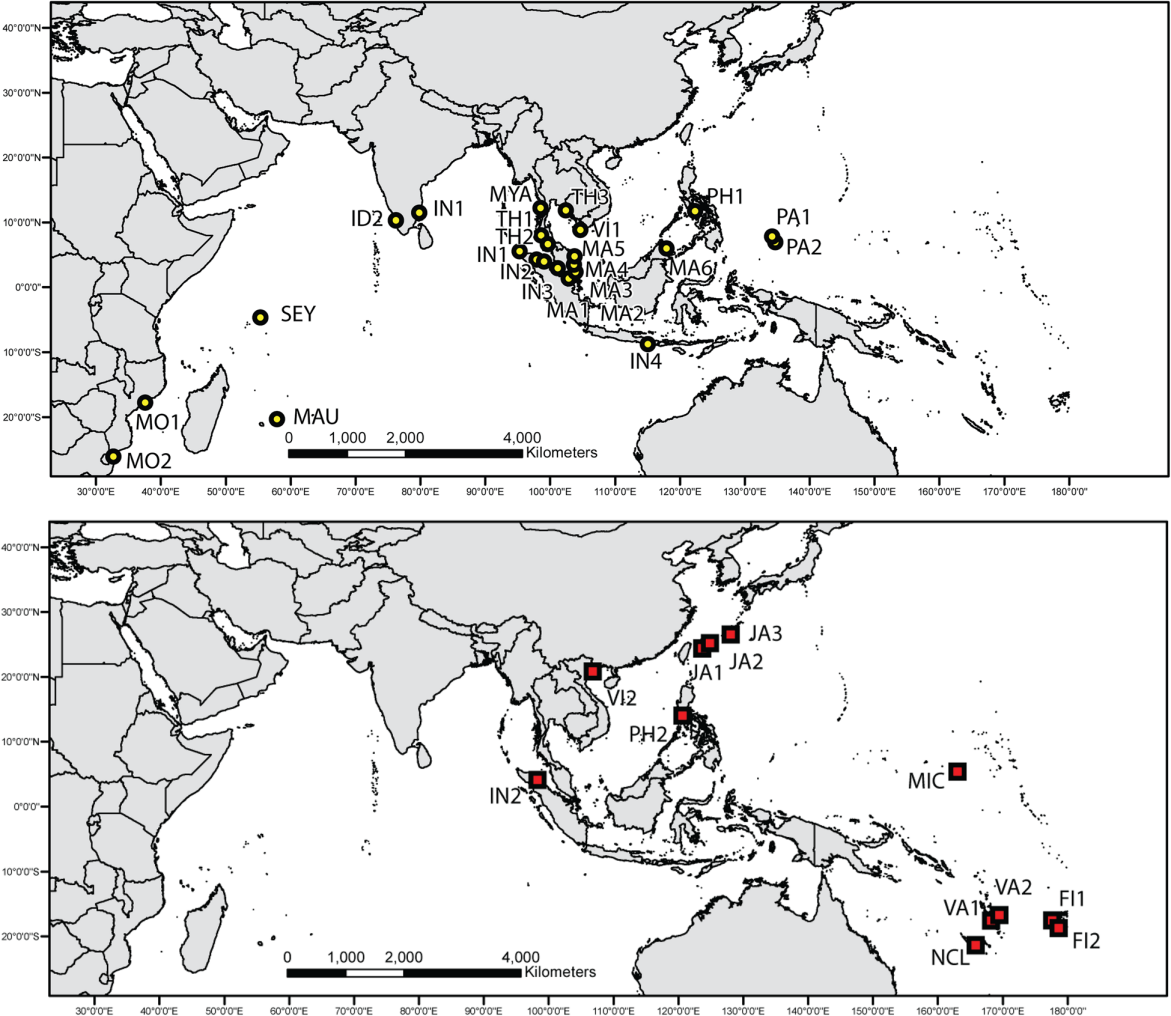
**Map depicting the location of study sites.** Yellow circles denote *Rhizophora mucronata* populations; red squares denote *Rhizophora stylosa* populations.

To resolve these problems on the genetic distinctiveness between *R. mucronata* and *R. stylosa*, we collected both species from their entire distribution range (Figure 1, Table 1) and genotyped them with 20 rapidly-mutating nuclear microsatellite (SSR) loci. The sample collection was conducted through an unprecedented level of international collaboration among mangrove scientists, whereby field identification and sampling in every site involved both local and international representatives of the cooperative network, using a standardized sampling protocol across a large geographical area. Specifically, we aim to determine the degree of genetic distinctiveness between these two species and their respective genetic structure.

**Table 1:** **Location information for all populations**

**Location**	**Country**	**Oceanic region**	**Pop. code**	**N**	**Latitude**	**Longitude**	**Voucher**
***R. mucronata***							
Quelimane	Mozambique	West Indian Ocean	MO1	34	17°52′58″S	36°51′40″E	OR10012802
Maupto	Mozambique	West Indian Ocean	MO2	37	25°51′00″S	32°41′44″E	TK11050101
Mahebourg	Mauritius	West Indian Ocean	MAU	32	20°25′41″S	57°43′34″E	OBY01012401
Mahe	Seychelles	West Indian Ocean	SEY	21	04°39′21″S	55°24′29″E	TK07101101
Cochin	India	Arabian Sea	ID1	27	09°59′29″N	76°14′07″E	TK07101201
Tamil Nadu	India	Bay of Bengal	ID2	32	11°25′52 N	79°47′38″E	TK07101401
Myeik	Myanmar	Andaman sea	MYA	30	12°23′53″N	98°34′11″E	TK12121401
Banda Aceh	Indonesia	Andaman sea*	IN1	33	05°34′33″N	95°19′08″E	TK07092705
Brandan	Indonesia	Andaman sea*	IN2	31	03°59′58″N	98°14′56″E	KT10073105
Jaring Halus	Indonesia	Strait of Malacca	IN3	27	03°56′22″N	98°33′46″E	KT10080201
Phuket	Thailand	Strait of Malacca	TH1	37	08°24′28″N	98°30′42″E	KT09012903
Palian	Thailand	Strait of Malacca	TH2	31	07°07′53″N	99°42′33″E	KT09012607
Klang	Thailand	Strait of Malacca	MA1	35	03°00′01″N	101°16′39″E	OR10012802
Benut	Malaysia	Strait of Malacca	MA2	39	01°36′22″N	103°16′17″E	TK11050101
Rompin	Malaysia	South China Sea	MA3	34	02°45′34″N	103°30′54″E	OBY01012401
Tanjung Lumpur	Malaysia	South China Sea	MA4	33	03°48′10″N	103°19′52″E	TK07101101
Paka	Malaysia	South China Sea	MA5	31	04°39′33″N	103°26′22″E	TK07101201
Nam Chiao	Thailand	South China Sea	TH3	32	12°09′57″N	102°28′36″E	TK07101401
Ca Mau	Vietnam	South China Sea	VI1	38	08°44′29″N	104°52′34″E	TK12121401
Sabah	Malaysia	South China Sea	MA6	30	05°56′19″N	118°03′10″E	TK07092705
Panay	Phillipines	South China Sea	PH1	35	11°47′28″N	122°13′41″E	KT10073105
Bali	Indonesia	Bali Sea	IN4	30	08°44′01″S	115°11′48″E	KT10080201
Airai	Palau	West Pacific Ocean	PA1	34	07°22′04″N	134°34′34″E	KT09012903
Karamadoo	Palau	West Pacific Ocean	PA2	23	07°30′12″N	134°32′09″E	KT09012607
***R. stylosa***							
Brandan	Indonesia	Strait of Malacca	IN2	22	03°59′58″N	98°14′56″E	OR10012802
Dong Rui	Vietnam	South China Sea	VI2	47	21°13′33″N	107°22′30″E	TK11050101
Lian Batangas	Phillipines	South China Sea	PH2	32	13°58′11″N	120°37′33″E	OBY01012401
Iriomote	Japan	East China Sea	JA1	30	24°24′09″N	123°46′28″E	TK07101101
Ishigaki	Japan	East China Sea	JA2	29	24°24′06″N	124°08′44″E	TK07101201
Okinawa	Japan	East China Sea	JA3	30	26°36′21″N	128°08′35″E	TK07101401
Kosrae	Micronesia	Northwest Pacific Ocean	MIC	31	05°20′55″N	163°01′09″E	TK12121401
Canala	New Caledonia	Southwest Pacific Ocean	NCL	28	21°30′26″S	165°58′09″E	TK07092705
Malatie	Vanuatu	Southwest Pacific Ocean	VA1	30	17°33′00″S	168°20′26″E	KT10073105
Maolapa	Vanuatu	Southwest Pacific Ocean	VA2	19	17°31′46″S	168°24′53″E	KT10080201
Lautoka	Fiji	Southwest Pacific Ocean	FI1	16	17°30′46″S	177°33′06″E	KT09012903
Mannikau	Fiji	Southwest Pacific Ocean	FI2	27	18°09′21″S	178°26′45″E	KT09012607

All specimens were identified by the collectors (one of the authors) designated by the initial at the first some letters of voucher information. Identification was confirmed by AWKS and TK.

*Although both Banda Aceh and Brandan are geographically located within the Strait of Malacca, Wee et al. [40] showed that these R. mucronata populations genetically clustered with the Andaman Sea populations. Hence we classified them under “Andaman Sea”.

N, number of individual per population.

## Results

### Genetic diversity

All loci were polymorphic, with the total number of alleles ranging from six to 16 per locus (mean = 10.25 alleles per locus) (see Additional file 1: Table S1 for genetic diversity parameters by locus). Low genetic diversity was detected in both species. The average observed heterozygosity (*H*_*O*_) across all populations was 0.108 and 0.097 for *R. mucronata* and *R. stylosa*, respectively (see Additional file 1: Table S2 for genetic diversity parameters by population). Levels of observed heterozygosity, expected heterozygosity and allelic richness were not significantly different between the two species (Unpaired *t* test, p > 0.05 for all comparisons).

A general heterozygote deficit was detected in both species; a significant level of inbreeding (*F*_*IS*_) was found in 85% and 67% of *R. mucronata* and *R. stylosa* populations, respectively (Additional file 1: Table S2). Deviation from Hardy-Weinberg Equilibrium (HWE) was significant in 235 out of 720 population–locus comparisons; seven of those were due to heterozygote excesses and the rest were associated with heterozygote deficits. All loci with heterozygote excesses were from the *R. mucronata* population in PA1. One quarter (25.8%) of the detected heterozygote deficits were associated with a particular population: 19 loci were from the *R. mucronata* population in PH1, and 18 and 15 loci were from the *R. stylosa* populations in MIC and FI2, respectively. Based on the null allele frequency estimated by FREENA, null alleles were potentially implicated (defined as null allele frequency > 0.10) in 30.1% of all population-locus combinations (see Additional file 1: Table S3 for null allele frequencies). Except for the four populations with heterozygote excess/deficit described above, the detection of potential null alleles was not associated with any locus or population.

### Inter-species genetic differentiation

The genetic diversity detected by the 20 loci employed in this study was sufficiently informative to reflect the genetic distinction between species. All loci harboured alleles that were unique to either one or both species. These species-specific alleles made up 57.6% of the total number of alleles in our data set. The proportion of species-specific alleles harboured by each locus ranged from 16.7% in RM107 to 75% in RM110 (see Additional file 1: Table S1).

The partitioning of genetic variation, as revealed by Analysis of Molecular Variance (AMOVA), was comparable when categorizing populations by species or by oceanic regions (Table 2). Between species, most of the genetic variation was partitioned among populations within species. Among regions, most of the genetic variation was partitioned among populations within oceanic regions.

**Table 2:** **AMOVA analysis comparing the genetic variation between species and among regions**

	**df**	**Variance component**	**Percentage variation**	**Φ-statistics**
**Between species**				
Between species	1	2.675	31.02	0.310***
Among populations within species	34	4.098	47.53	0.689***
Within populations	2175.4	1.850	21.45	0.785***
**Among oceanic regions**				
Among regions	6	2.414	31.96	0.320***
Among populations within regions	29	3.256	43.11	0.634***
Within populations	2175.4	1.883	24.93	0.751***
**Within** ***R. mucronata***				
Among regions	4	2.477	40.48	0.405***
Among populations within regions	18	1.899	31.04	0.522***
Within populations	1395.5	1.743	28.48	0.715***
**Within** ***R. stylosa***				
Among regions	3	3.051	45.82	0.458***
Among populations within regions	7	2.004	30.11	0.556***
Within populations	605.8	1.602	24.07	0.759***

df; degree of freedom, ***; significant at the P < 0.001 level.

**Figure 2:**
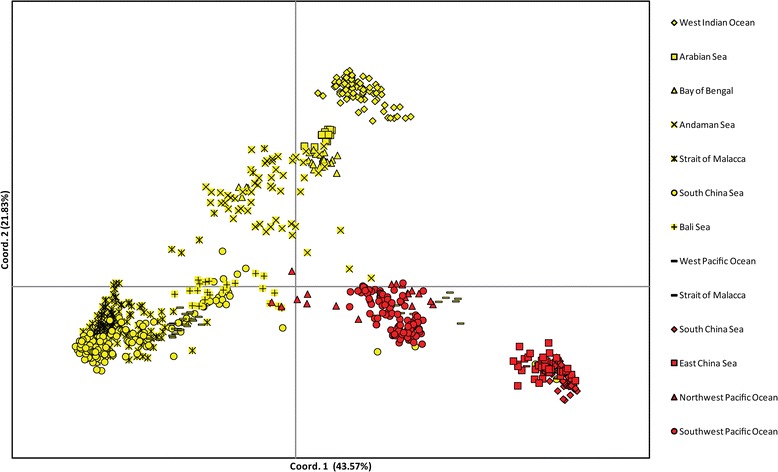
**PCoA scatter plot showing the genetic distance among individuals according to oceanic region.** The percentage of total variation attributed to each axis is as indicated. *Rhizophora mucronata* individuals are indicated with yellow markers; *Rhizophora stylosa* individuals are indicated with red markers.

**Figure 3:**
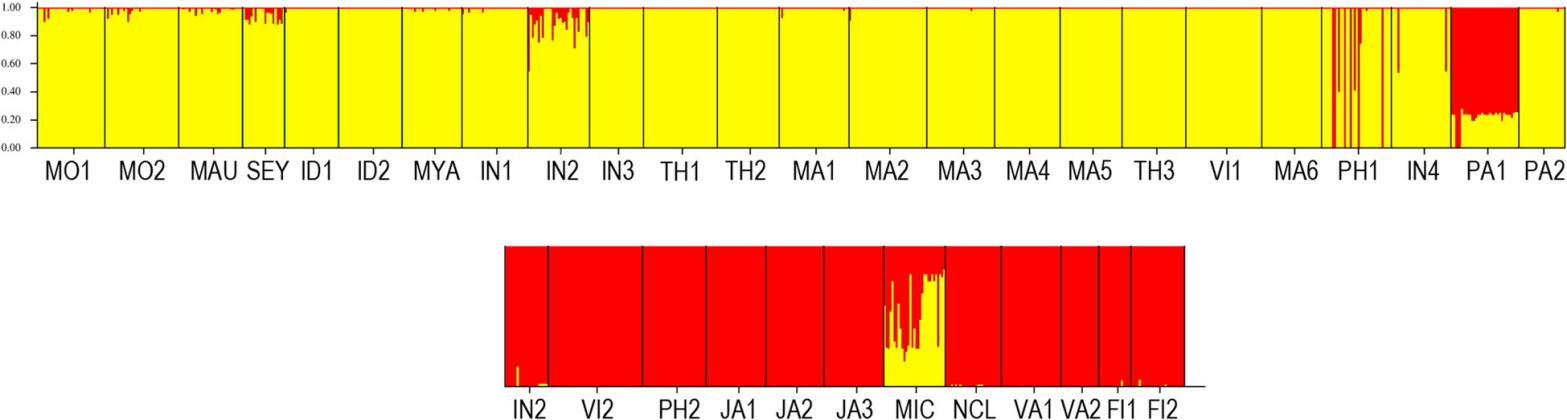
**Structure bar plots showing the assignment of individuals into two distinct genetic clusters (K = 2).** *Rhizophora mucronata* individuals are indicated in yellow; *Rhizophora stylosa* individuals are indicated in red markers.

We detected an overall strong genetic structure across all populations, with significant genetic differentiation (*F*_ST_) estimated at 0.737 averaging all loci and populations (p < 0.001). All pairwise population genetic differentiation was significant at the p < 0.001 level, except between adjacent *R. mucronata* populations MA1 and MA2 (pairwise *F*_ST_ = 0.013, p > 0.05), and *R. stylosa* populations VA1 and VA2 (pairwise *F*_ST_ = 0.044, p > 0.05). Pairwise *F*_ST_ estimates are listed in Additional file 1: Table S4. The PCoA results demonstrated a clear genetic differentiation between *R. mucronata* and *R. stylosa*, as well as among oceanic region in each species (Figure 2). There was no overlap between species, except for (1) several *R. mucronata* individuals from the South China Sea region observed in the *R. stylosa* clusters, and (2) an overlap of *R. mucronata* individuals from Bali Sea (IN4) with *R. stylosa* individuals from northwest Pacific Ocean (MIC). Model-based individual assignment via *STRUCTURE* was in agreement with the PCoA results. We found strong support for two genetic clusters among our samples that generally corresponded to the respective species (Figure 3). All *R. mucronata* individuals had > 90% of inferred ancestry from the same genetic cluster except for several individuals in populations SEY, IN2, PH1 and IN4. *R. mucronata* individuals from PA1 had more than 50% inferred ancestry from the *R. stylosa* genetic cluster, hence may represent putative hybrids between the two species. Similarly, mixed inferred ancestry was also found in *R. stylosa* individuals from MIC.

**Figure 4:**
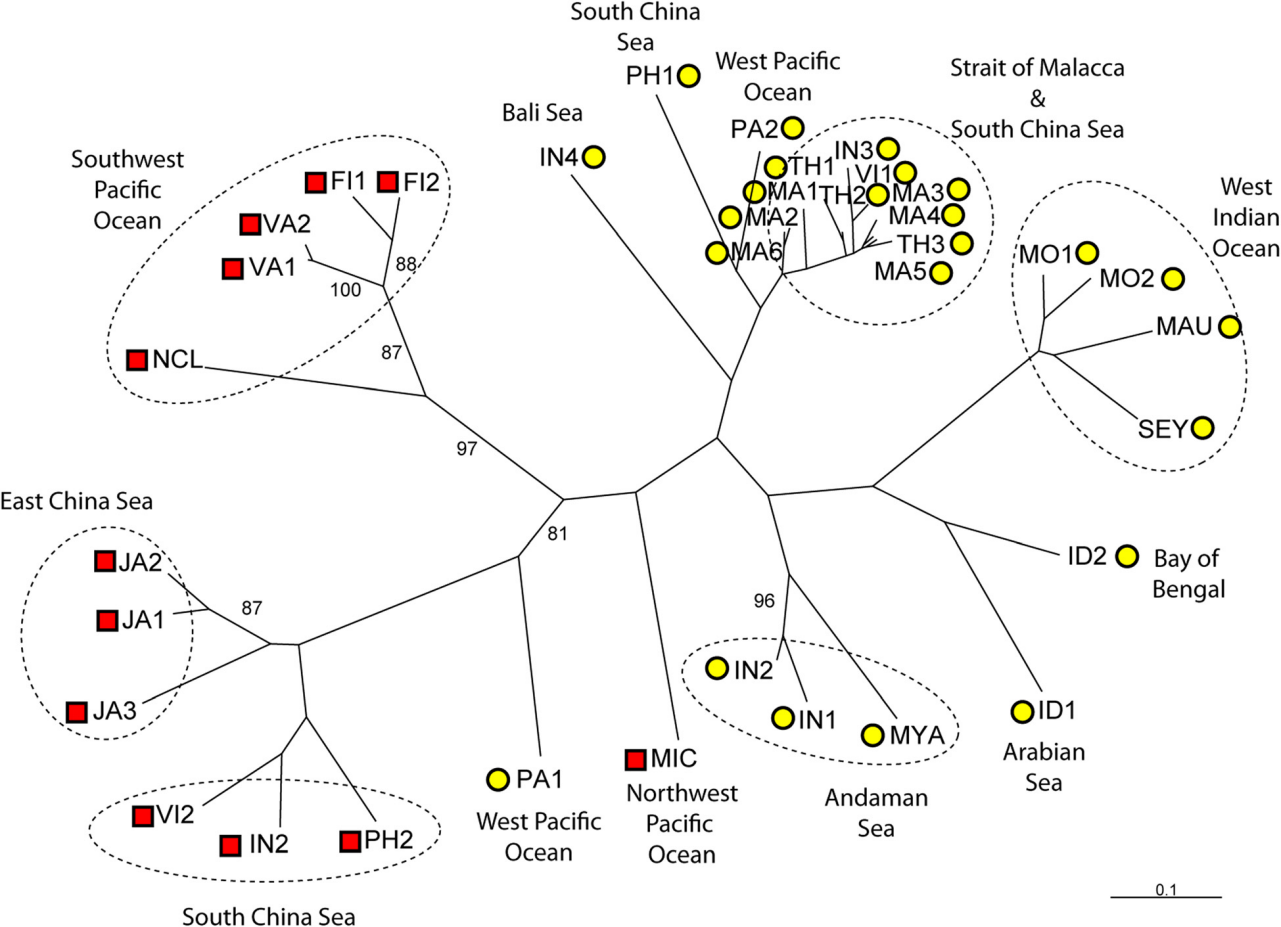
**Neighbour-joining (NJ) tree showing the relationships among populations.** Dotted ellipses outline the clusters of populations belonging to the same oceanic region. Yellow circles denote *Rhizophora mucronata* populations; red squares denote *Rhizophora stylosa* populations.

The relationships between populations in the NJ tree supported the findings from PCoA and *STRUCTURE*. Genetic clustering of populations was in concordance with their respective species and oceanic region (Figure 4). With the exception of *R. mucronata* population PA1 and *R. stylosa* population MIC, of which individuals were estimated to have mixed ancestry by *STRUCTURE*, the NJ tree supported a genetic distinction between *R. mucronata* and *R. stylosa*.

### Intra-species genetic differentiation

**Figure 5:**
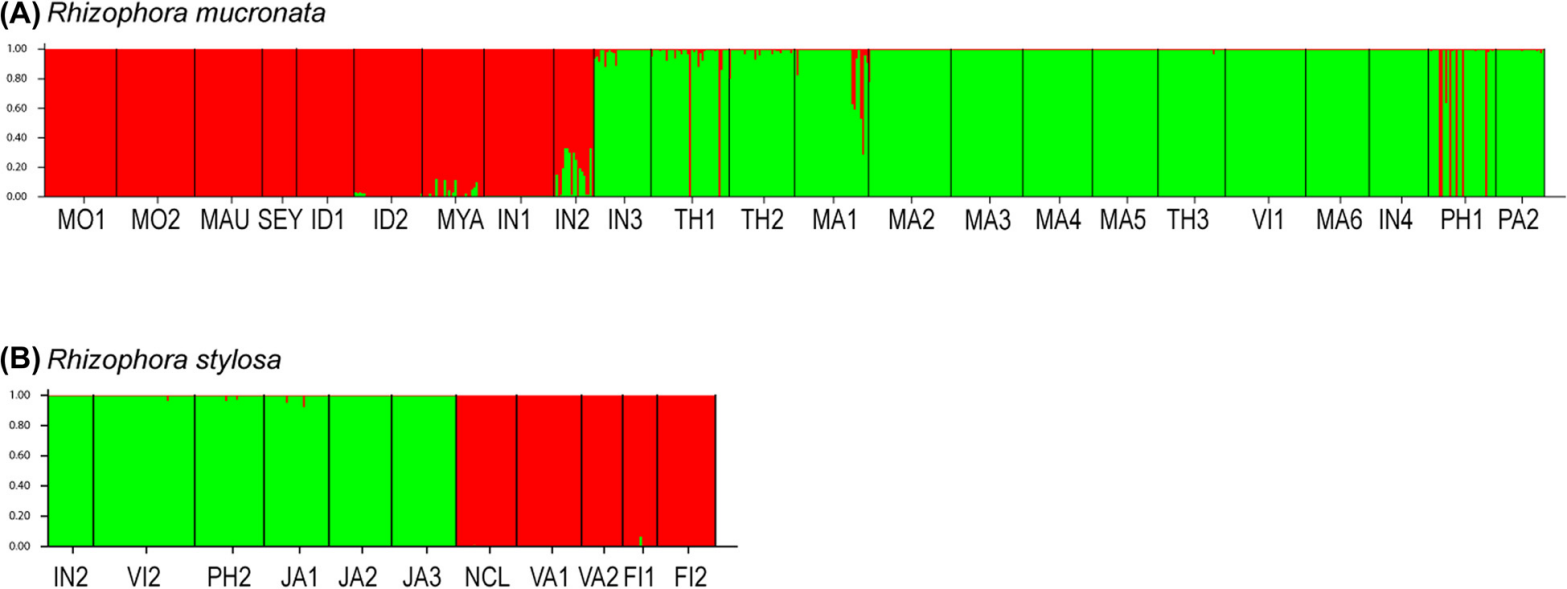
**Structure bar plots showing the assignment of individuals into two distinct genetic clusters (K = 2) for both (A)** ***Rhizophora mucronata*** **and (B)** ***Rhizophora stylosa*** **individuals.**

**Figure 6:**
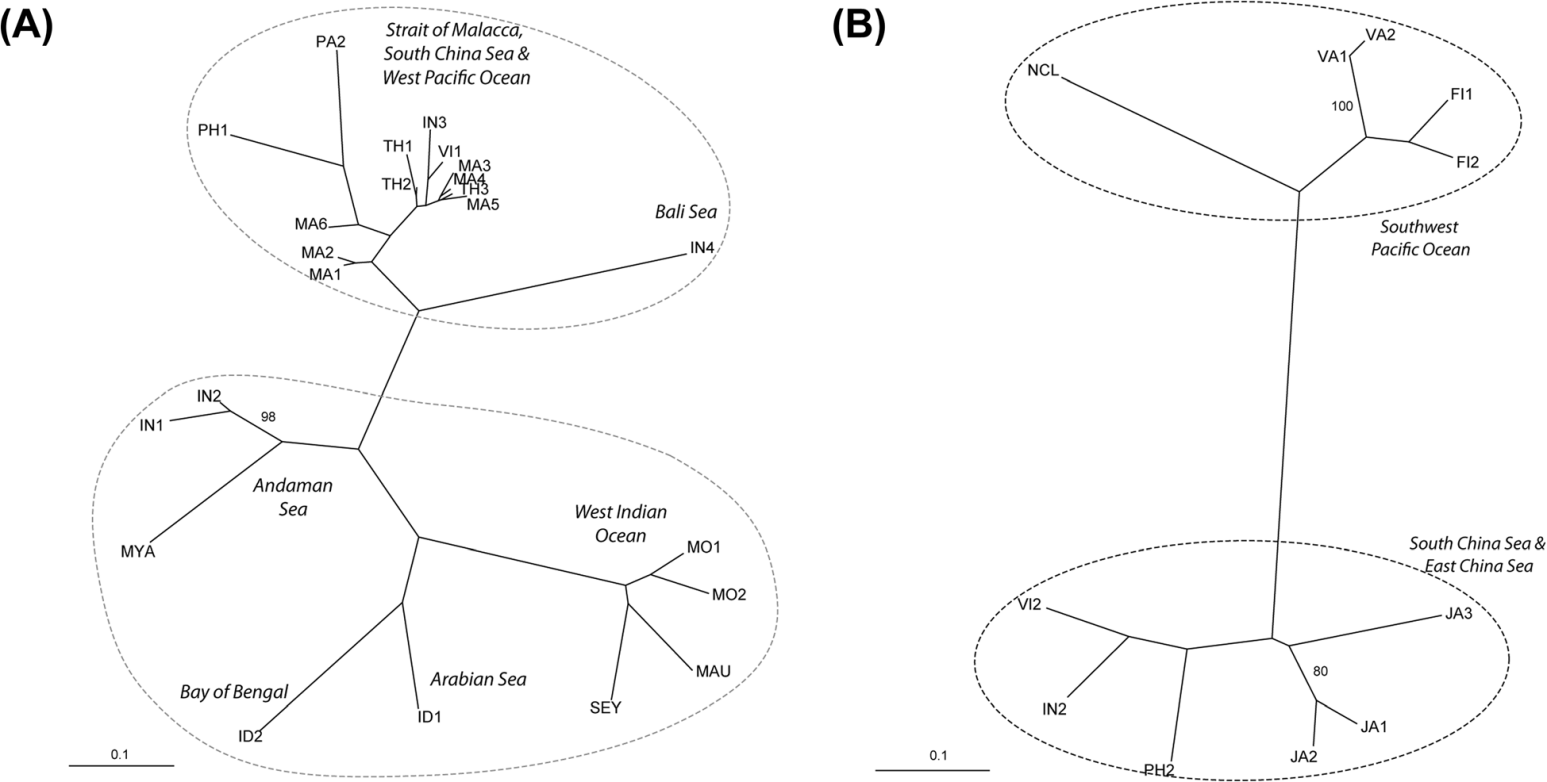
**Neighbour-joining (NJ) tree showing the relationships among populations for (A)** ***Rhizophora mucronata*** **and (B)** ***Rhizophora stylosa*** **.** The oceanic region of each population cluster is indicated in italics. Dotted ellipses outline the clusters as denoted by the STRUCTURE analysis.

STRUCTURE analysis with pure individuals (after removing both putative hybrids and possibly misidentified individuals) supported two genetic clusters (K = 2) for both *R. mucronata* and *R. stylosa* (Figure 5). In *R. mucronata*, the two genetic clusters were: (1) populations from West Indian Ocean, Arabian Sea, Bay of Bengal and Andaman Sea, and (2) populations from Malacca Strait, South China Sea, Bali Sea and West Pacific Ocean (Figure 6A). Low level of admixture was detected in *R. mucronata* populations from the Andaman Sea and Malacca Strait. Two genetic clusters were also detected in *R. stylosa*; the clustering pattern was more conspicuous than that of *R. mucronata*. A strong genetic break separated populations in the South China Sea and East China Sea from populations in the Southwest Pacific Ocean (Figure 6B). The genetic breaks were constantly supported with increasing number of clusters in STRUCTURE analyses. AMOVA analysis revealed that within each species, most of the genetic variation was partitioned among regions (40.48% and 45.82% for *R. mucronata* and *R. stylosa*, respectively) (Table 2).

## Discussion

### Genetic distinctiveness between *R. mucronata* and *R. stylosa*

Our study provides strong evidence of the genetic distinctiveness between *R. mucronata* and *R. stylosa*. Even though the genetic proximity of these two species is unquestionable—as all 20 SSR loci were successfully amplified in both species—we are confident of the genetic distinctiveness between both species based on two lines of evidences. First, a large proportion of alleles detected in our study were unique to each species. This indicated that the detected genetic divergence was not merely a difference in allele frequency, which may be prone to the effects of population processes such as bottlenecks and genetic drift [30]. Second, the genetic assignment of individuals by species was consistent with our field identification, even in populations from Southeast Asia where the distribution ranges of both species overlap (e.g. in IN2 where both *R. mucronata* and *R. stylosa* were collected). Therefore, these two species remained as distinct genetic entities even in close geographical proximity, either via reproductive isolation or the fixation of alleles resulting from historical vicariance.

Due to the fine resolution afforded by polymorphic SSR markers, our study also detected admixed genotypes in *R. mucronata* population PA1 and *R. stylosa* population MIC despite a clear inter-species genetic divergence in all other populations. As most of the individuals in these two populations tend to have admixed genetic characteristics despite the clear morphological assignment to one species, we inferred that hybridization and continuous introgression might be occurring in these two populations. Genetic evidence recently confirmed the presence of hybrids between *R. mucronata* and *R. stylosa* [18,31], even though morphological intermediates and the lack of complete ecological reproductive isolation (in flowering phenology and niche specialization) between the two species have long suggested it [22]. Therefore, the presence of potentially hybrid-derived lineages between *R. mucronata* and *R. stylosa*, though few, raise doubts on the integrity of these two species and the most appropriate species boundary applicable to them.

Hybridization or introgression between two genetically distinct species is not uncommon among coastal organisms and is usually attributed to a recent overlap of distributional ranges following historical geographical separation [32,33]. The substantial geological age of the genus *Rhizophora* (estimated at 50 million years) [22] and its coastal distribution presented opportunities for repeated population contraction and expansion—and consequently reproductive isolation and introgression—among its species during the glacial-interglacial cycles. If indeed the dispersal centers of ancestral *R. mucronata* and *R. stylosa* were as postulated in East Africa and Australasia, respectively [22], then the present interglacial period would have brought these two species into contact. Hence, hybridization or introgression in sympatric populations would be possible. Previous studies on coral reef fishes reported a marine hybrid hotspot at Christmas and Cocos Islands, located at the Indo-Pacific biogeographic border [34]. Our data, coupled with that of Ng *et al.* [18], suggested that the hybrid zones for *R. mucronata* and *R. stylosa* might be much wider and located further eastward, between Southeast Asia and Micronesia.

### Phylogeography of *R. mucronata* and *R. stylosa*

By excluding genetically mixed individuals from the analysis, our study was able to provide a more definitive representation of the intra-species genetic structure. The phylogeography of *R. mucronata* and *R. stylosa*, investigated independently after removing putative hybrids, demonstrated a close association between the genetic structure and oceanic region. This supports the general genetic patterns in marine and coastal species whereby ocean currents act to maintain gene flow within an oceanic region and prevent gene flow between oceanic regions [35].

In *Rhizophora mucronata*, a strong divergence was detected between populations from the Indian Ocean region (Indian Ocean and Andaman Sea) and the Pacific Ocean region (Malacca Strait, South China Sea and Northwest Pacific Ocean). The dichotomous genetic differentiation into Indian and Pacific Ocean lineages have been reported in other mangrove species [36,37] and coastal fauna [38,39], and was attributed to the role of Sundaland as a land barrier during past glaciations periods. *R. mucronata* population IN4, located at the boundary between the two oceanic regions, was an exception to this dichotomous division. Our results showed that IN4 was included in the Pacific Ocean genetic cluster, thus indicating that it either shared the same ancestry as, or had maintained frequent gene flow with, the other populations from the Pacific Ocean. The genetic break we detected at the northern boundary of the Malacca Strait concurred with an earlier study involving a smaller geographical coverage of Southeast Asia [40]. An analysis of regional oceanic circulation patterns suggested that contemporary oceanic currents may act as a cryptic barrier to gene flow that prevents admixture across this genetic boundary [40].

A dichotomous divergence was also observed in *R. stylosa*, though the genetic break was located more eastward, between populations from South and East China Sea and populations from the Southwest Pacific Ocean. This was supported by a previous study which found a genetic disjunction between populations from the Malay Peninsula and Japan [41]. The location of the genetic break coincided with complex surface currents at the western equatorial Pacific Ocean—aptly described as a “water mass crossroads” [42]—that may have prevented gene flow between the two oceanic regions. Similar north–south genetic divergence observed around the equator was also observed in the phylogeography of Atlantic coral fishes, indicating that oceanography has a substantial influence on the genetic structure of sea-dispersed organisms [43]. The detection of this genetic break together with the presence of putative hybrids in the MIC (Kosrae) population calls for more detailed investigation on the gene flow among *R. stylosa* populations from the Pacific islands located around the equator, as well as cross-comparisons with other sympatric mangroves, such as *Bruguiera gymnorhiza.*

Our findings differed from that of a previous investigation using chloroplast DNA and nuclear ribosomal DNA, which showed that *R. apiculata*, *R. mucronata* and *R. stylosa* shared similar genetic structure [24]. Lo *et al.* [24] reported that a common genetic break—separating the populations into one cluster from Southeast Asia and Sri Lanka and another from Africa, Australia and the Pacific—was found in all three species. As both studies employed molecular markers with different mutation properties, it is possible that the data sets represent genetic patterns across different ecological and evolutionary time scales. Our findings, generated by nuclear SSR markers, tend to demonstrate more contemporary gene flow while those from Lo *et al.* [24] represented historical gene flow that may have dated back to the Oligocene-Miocene boundary circa 29–24 Ma. Future studies, with finer-resolution molecular dating and additional sampling at the genetic boundaries will be able to fill in the gap between both studies and further elucidate the colonization pathways and species boundaries between *R. mucronata* and *R. stylosa*.

The genetic clusters we identified in *R. mucronata* and *R. stylosa* provide a basis for the definition of ESUs in these species. Even though the exact levels of molecular phylogenetic distinctiveness required for the definition of ESUs are still debatable [44,45], the two genetic clusters found in each species were geographically discrete, suggesting prolonged genetic and physical isolation. Hence, populations in these two clusters should be considered as distinct ESUs. Our genetic data can be further combined with morphological measurements [22] to verify the adaptive significance of the observed divergence in allele frequencies. We recommend that these genetic clusters should be managed separately and care should be taken to avoid artificial transplantation of individuals from a different cluster.

### Low genetic diversity and heterozygosity

In this study, low levels of genetic diversity and heterozygosity were widespread in *R. mucronata* and *R. stylosa* populations. Excessive homozygosity has been shown in *Rhizophora* [41], and is common in mangroves and mangrove associates, and may be attributable to low genetic diversity at range limits [46,47], the presence of null alleles [48,49] or inbreeding [50-52]. Since the studied populations were from the entire species distribution range, the observed heterozygote deficit is unlikely to result from low genetic diversity at the range limit. Our data indicated that null alleles might be present at several loci (frequency < 0.2) and may have led to heterozygote deficit. However, null allele frequencies can be overestimated in inbred populations that are not under HWE [53]. Inbreeding and self-compatibility are expected to be higher in mangroves than in other tropical plants because these are traits that facilitate the colonization of distant locations [54]. Indeed, *Rhizophora* species have been shown to be self-compatible [27,55,56], possessing flowers that are mainly wind-pollinated but with facultative pollination by small insects [27,57]. For example, pollinator limitation is common in *R. stylosa*, which exhibits a typical fertilization rate of only 3-4% under natural conditions [57,58]. Thus, pollinator limitation, which often leads to selfing [59], may be widespread in *Rhizophora*. Inbreeding in *R. mucronata* and *R. stylosa* could be similarly expected in naturally fragmented mangrove habitats, as fragmentation reduces pollen availability and the number of pollen donor in wind-pollinated plants [60,61]. Since the excess of homozygotes is in concordance with the biology of *Rhizophora*, we interpret this as a result of inbreeding rather than the presence of null alleles.

## Conclusions

Our study represents the first population genetic studies covering the entire distributional range of the species complex *R. mucronata* and *R. stylosa*. By employing a suite of 20 informative nuclear SSR markers, we demonstrated the general genetic distinctiveness of *R. mucronata* and *R. stylosa*, and potential hybridization or introgression between them. Since inter-species gene flow was implicated, we investigated the population genetics of each species without the putative hybrids, and found strong genetic structure between oceanic regions in both *R. mucronata* and *R. stylosa*. Both species showed a dichotomous genetic divergence among their respective populations. Even though the locations of the genetic break were different for each species, they coincided with the boundaries of oceanic currents, thus suggesting that oceanic circulation patterns might have acted as a cryptic barrier to gene flow.

Our findings have important implications on the conservation of mangroves, especially relating to replanting efforts and the definition of ESUs in *Rhizophora* species. Previous studies on Californian seagrass revealed that unintentional anthropogenic mixing of two genetically distinct species in a transplantation effort might have promoted recent hybridization and introgression between them [62]. This highlighted the need to recognize the genetic distinctiveness of closely-related species, determine their respective genetic structure, and avoid artificially promoting hybridization in mangrove restoration programmes. Hence, our results serve as the foundation for the conservation genetics of *R. mucronata* and *R. stylosa* by outlining their respective genetic structure and identifying geographical areas that require further investigations.

## Methods

### Population sampling and genotyping

From 2008 to 2012, *R. mucronata* and *R. stylosa* samples were collected from a total of 24 and 12 populations, respectively, from the entire Indo-West Pacific region (16–47 individuals per population) (Table 1). Sample collection was conducted under the Research Network for Conservation Genetics of Mangrove. To ensure consistency, species identification was performed by one local and one international representative of the network according to morphological characteristics that were previously reported to be useful in distinguishing both species [15,22] and local knowledge of species distribution. Voucher specimens used for the identification were deposited in URO (the University of Ryukyus). A leaf sample was collected from each individual and dried in silica gel. Genomic DNA was extracted using a modified CTAB method [63].

Twenty SSR loci were employed in this study. Twelve were developed for *R. mucronata*: RM102, RM103, RM107, RM110, RM111, RM112, RM114, RM116, RM121 [64], RMu21, RMu35 and RMu54 [65]; and eight were developed for *R. stylosa*: Rhst01, Rhst02, Rhst11, Rhst13, Rhst15 [66], RS19, RS59 and RS78 [67]. All loci were genotyped using fluorescent-labeled primers with the following dye-primer combinations – 6-FAM: RM102, RM107, RM110, Rhst01, Rhst02, Rhst11, and Rhst15; VIC: RM103, RM114, RM116, RMu35 and RS19; NED: RM121, RMu21 and RMu54; PET: RM111, RM112, Rhst13, RS59 and RS78. The polymerase chain reaction (PCR) conditions were: initial denaturation 5 min at 94°C; 35 cycles of 45 s at 95°C, 45 s at 50°C, 45 s at 72°C; final elongation of 10 min at 72°C. Total reaction volume was 10 μL, of which 1.5 μL was DNA. PCR was conducted with iTaq DNA polymerase (i-DNA Biotechnology, Singapore). PCR products were run on an ABI 3130xl automated sequencer with the GeneScan-600 LIZ size standard and analysed using Genemapper 4.1 (Applied Biosystems, Grand Island, NY, USA).

### Genetic diversity and heterozygote deficiency

To estimate the genetic diversity within population, expected heterozygosity (*H*_E_) and observed heterozygosity (*H*_O_) were calculated using GenAlEx 6.5 software [68]. The software fstat 2.9.3.2 [69] was used to compute the allelic richness (*A*_R_) and heterozygote deficiency (estimated by *F*_IS_ and assessed at the P < 0.05 significance level). Allelic richness was rarefied to the minimum sample size of 16 individuals.

Null allele frequencies were estimated for each locus and population with FREENA [70], using the expectation maximization algorithm of [71]. Deviations from HWE were tested for each locus and population by an exact test using Genepop 3.4 [72].

### Inter-species genetic differentiation

The pattern for genetic differentiation was visualized via one individual-based analysis, the Principal Coordinate Analysis (PCoA), and two population-based analyses, an unrooted consensus neighbor-joining (NJ) tree and a Bayesian model-based clustering method implemented in the software STRUCTURE [73]. PCoA was performed using GenAlEx v6.5 software [67] based on the mean genotypic distance between all individual pairs of both species. The NJ tree was generated with POPULATIONS v.1.2.31 [74] using Nei et al.’s (1983) *D*_A_ as an estimator for the genetic distance between populations [75]. The STRUCTURE clustering analysis was conducted by employing the admixture model, with 20 runs for each number of subpopulations (*K*), from K = 1 to K = 15. Each run consisted of 10^6^ replicates of the Markov chain Monte Carlo (MCMC) after a burn-in of 10^5^ replicates. The most likely number of population clusters was estimated by the ∆K parameter [76] using the Structure Harvester online program [77].

AMOVA analysis was conducted to determine the partitioning of genetic variation under two scenarios: (1) populations were grouped according to species; and (2) populations were grouped according to oceanic region regardless of species (refer to Table 1 for oceanic region categories). The AMOVA was conducted in Arlequin with a 10,000 permutations [78].

### Genetic differentiation within-species

For each species, population differentiation (*F*_ST_) averaged across all loci, and pairwise *F*_ST_ estimates between all population pairs [79] were calculated using fstat 2.9.3.2. [69].

Based on results from inter-species STRUCTURE analysis, putative hybrids (genetically mixed individuals) between two species were identified and removed from the following analysis. The hybrids were defined as individuals with < 90% of its genotype having an inferred ancestry from either species when K = 2 for the inter-species STRUCTURE analysis. The genetic clusters were determined using STRUCTURE [73] with the same run parameters as the inter-species analysis.

### Availability of supporting data

The data set supporting the results of this article is available in the Dryad repository [80], http://datadryad.org/resource/doi:10.5061/dryad.42711/1.

## Additional file

Additional file 1: Table S1. Genetic diversity parameters of the twenty microsatellite loci employed in this study. **Table S2.** Genetic diversity parameters of sampled populations. **Table S3.** Null allele frequency as estimated by FREENA for each population-locus comparison. **Table S4.** Pairwise FST estimates between all population pairs.

## References

[1] Duke NC, Meynecke JO, Dittmann S, Ellison AM, Anger K, Berger U (2007). A World Without Mangroves?. Science.

[2] Victor S, Golbuu Y, Wolanski E, Richmond R (2004). Fine sediment trapping in two mangrove-fringed estuaries exposed to contrasting land-use intensity, Palau, Micronesia. Wetl Ecol Manag.

[3] Bouillon S, Borges AV, Castañeda‐Moya E, Diele K, Dittmar T, Duke NC (2008). Mangrove production and carbon sinks: a revision of global budget estimates. Global Biogeochemical Cycles.

[4] Robertson A, Duke N (1987). Mangroves as nursery sites: comparisons of the abundance and species composition of fish and crustaceans in mangroves and other nearshore habitats in tropical Australia. Mar Biol.

[5] Kathiresan K, Rajendran N (2005). Coastal mangrove forests mitigated tsunami. Estuar Coast Shelf Sci.

[6] Tanaka N, Sasaki Y, Mowjood M, Jinadasa K, Homchuen S (2007). Coastal vegetation structures and their functions in tsunami protection: experience of the recent Indian Ocean tsunami. Landsc Ecol Eng.

[7] Valiela I, Bowen JL, York JK (2001). Mangrove Forests: One of the World’s Threatened Major Tropical Environments. Bioscience.

[8] Barbier EB (2006). Natural barriers to natural disasters: replanting mangroves after the tsunami. Front Ecol Environ.

[9] Triest L (2008). Molecular ecology and biogeography of mangrove trees towards conceptual insights on gene flow and barriers: A review. Aquat Bot.

[10] Chen L, Wang W, Zhang Y, Lin G (2009). Recent progresses in mangrove conservation, restoration and research in China. J Plant Ecol.

[11] Sheue C-R, Liu H-Y, Tsai C-C, Yang Y-P (2010). Comparison of Ceriops pseudodecanda sp. nov. (Rhizophoraceae), a new mangrove species in Australasia, with related species. Botanical Studies.

[12] Takayama K, Tamura M, Tateishi Y, Webb EL, Kajita T (2013). Strong genetic structure over the American continents and transoceanic dispersal in the mangrove genus *Rhizophora* (Rhizophoraceae) revealed by broad-scale nuclear and chloroplast DNA analysis. Am J Bot.

[13] Su G, Huang Y, Tan F, Ni X, Tang T, Shi S (2007). Conservation genetics of *Lumnitzera littorea* (Combretaceae), an endangered mangrove, from the Indo-West Pacific. Mar Biol.

[14] Tan F, Huang Y, Ge X, Su G, Ni X, Shi S (2005). Population genetic structure and conservation implications of *Ceriops decandra* in Malay Peninsula and North Australia. Aquatic Botany.

[15] Tomlinson PB (1986). The Botany of Mangroves.

[16] Kathiresan K (1995). *Rhizophora annamalai*: a new species of mangroves. Environ Ecol.

[17] Parani M, Rao CS, Mathan N, Anuratha CS, Narayanan KK, Parida A (1997). Molecular Phylogeny of mangroves III Parentage analysis of a Rhizophora hybrid using random amplified polymorphic DNA and restriction fragment length polymorphism markers. Aquat Bot.

[18] Ng WL, Chan HT, Szmidt AE (2013). Molecular identification of natural mangrove hybrids of Rhizophora in Peninsular Malaysia. Tree Genetics Genomes.

[19] Macintosh D, Ashton E, Havanon S (2002). Mangrove rehabilitation and intertidal biodiversity: a study in the Ranong mangrove ecosystem, Thailand. Estuar Coast Shelf Sci.

[20] Field CD (1999). Rehabilitation of Mangrove Ecosystems: An Overview. Mar Pollut Bull.

[21] Salmo SG, Lovelock C, Duke NC (2013). Vegetation and soil characteristics as indicators of restoration trajectories in restored mangroves. Hydrobiologia.

[22] Duke N, Lo E, Sun M (2002). Global distribution and genetic discontinuities of mangroves - emerging patterns in the evolution of Rhizophora. Trees Structure Function.

[23] Inomata N, Wang X-R, Changtragoon S, Szmidt AE (2009). Levels and patterns of DNA variation in two sympatric mangrove species, *Rhizophora apiculata* and *R. mucronata* from Thailand. Genes Genetic Syst.

[24] Lo EY, Duke NC, Sun M (2014). Phylogeographic pattern of Rhizophora (Rhizophoraceae) reveals the importance of both vicariance and long-distance oceanic dispersal to modern mangrove distribution. BMC Evol Biol.

[25] Spalding MD, Kainuma M, Collins L (2010). World Atlas of Mangrove Earthscan.

[26] Setyawan AD, Ulumuddin YI, Ragavan P (2014). Review: Mangrove hybrid of Rhizophora and its parental species in Indo-Malayan region.

[27] Kondo K, Nakamura T, Tsuruda K, Saito N, Yaguchi Y (1987). : Pollination in *Bruguiera gymnorrhiza* and *Rhizophora mucronata* (Rhizophoraceae) in Ishigaki Island, The Ryukyu Islands, Japan. Japan Biotropica.

[28] Okimoto Y, Nose A, Katsuta Y, Tateda Y, Agarie S, Ikeda K (2007). Gas exchange analysis for estimating net CO_2_ fixation capacity of mangrove (*Rhizophora stylosa*) forest in the mouth of river Fukido, Ishigaki Island, Japan. Plant Production Sci.

[29] Lo E (2010). Testing hybridization hypotheses and evaluating the evolutionary potential of hybrids in mangrove plant species. J Evol Biol.

[30] Luikart G, Cornuet JM (1998). Empirical evaluation of a test for identifying recently bottlenecked populations from allele frequency data. Conserv Biol.

[31] Ng WL, Szmidt AE (2015). Introgressive hybridization in two Indo-West Pacific *Rhizophora* mangrove species, *R. mucronata* and *R. stylosa*. Aquat Bot.

[32] Baumel A, Ainouche M, Bayer R, Ainouche A, Misset M (2002). Molecular Phylogeny of Hybridizing Species from the Genus Spartina Schreb. (Poaceae). Mol Phylogenet Evol.

[33] Van Herwerden L, Choat J, Dudgeon C, Carlos G, Newman S, Frisch A (2006). Contrasting patterns of genetic structure in two species of the coral trout *Plectropomus* (Serranidae) from east and west Australia: Introgressive hybridisation or ancestral polymorphisms. Mol Phylogenet Evol.

[34] Hobbs J-PA, Frisch AJ, Allen GR, Van Herwerden L (2009). Marine hybrid hotspot at Indo-Pacific biogeographic border. Biol Lett..

[35] Kool JT, Paris CB, Barber PH, Cowen RK (2011). Connectivity and the development of population genetic structure in Indo-West Pacific coral reef communities. Glob Ecol Biogeogr.

[36] Su G-H, Huang Y-L, Tan F-X, Ni X-W, Tang T, Shi S-H (2006). Genetic variation in *Lumnitzera racemosa*, a mangrove species from the Indo-West Pacific. Aquat Bot.

[37] Huang Y, Tan F, Su G, Deng S, He H, Shi S (2008). Population genetic structure of three tree species in the mangrove genus *Ceriops* (Rhizophoraceae) from the Indo West Pacific. Genetica.

[38] Barber P, Palumbi S, Erdmann M, Moosa M (2002). Sharp genetic breaks among populations of *Haptosquilla pulchella* (Stomatopoda) indicate limits to larval transport: patterns, causes, and consequences. Mol Ecol.

[39] Benzie JAH (1999). Major Genetic Differences between Crown-of-Thorns Starfish (*Acanthaster planci*) Populations in the Indian and Pacific Oceans. Evolution.

[40] Wee AK, Takayama K, Asakawa T, Thompson B, Onriza, Sungkaew S (2014). Oceanic currents, not land masses, maintain the genetic structure of the mangrove Rhizophora mucronata Lam. (Rhizophoraceae) in Southeast Asia. J Biogeogr.

[41] Ng WL, Onishi Y, Inomata N, Teshima KM, Chan HT, Baba S (2015). Closely related and sympatric but not all the same: genetic variation of Indo-West Pacific Rhizophora mangroves across the Malay Peninsula. Conservation Genetics.

[42] Fine RA, Lukas R, Bingham FM, Warner MJ, Gammon RH (1994). The western equatorial Pacific: A water mass crossroads. J Geophysical Res: Oceans (1978–2012).

[43] Muss A, Robertson DR, Stepien CA, Wirtz P, Bowen BW (2001). Phylogeography of Ophioblennius: the role of ocean currents and geography in reef fish evolution. Evolution.

[44] Moritz C (1994). Defining ‘Evolutionarily Significant Units’ for conservation. Trends Ecol Evol.

[45] Waples R (1991). Pacific Salmon, *Oncorhynchus* spp., and the Definition of “Species” Under the Endangered Species Act. Mar Fish Rev.

[46] Arnaud-Haond S, Teixeira S, Massa SI, Billot C, Saenger P, Coupland G (2006). Genetic structure at range edge: low diversity and high inbreeding in Southeast Asian mangrove (*Avicennia marina*) populations. Mol Ecol.

[47] Maguire TL, Saenger P, Baverstock P, Henry R (2000). Microsatellite analysis of genetic structure in the mangrove species *Avicennia marina* (Forsk.) Vierh. (Avicenniaceae). Mol Ecol.

[48] Arbeláez-Cortes E, Castillo-Cárdenas M, Toro-Perea N, Cárdenas-Henao H (2007). Genetic structure of the red mangrove (*Rhizophora mangle* L.) on the Colombian Pacific detected by microsatellite molecular markers. Hydrobiologia.

[49] Takayama K, Tateishi Y, Murata JIN, Kajita T (2008). Gene flow and population subdivision in a pantropical plant with sea-drifted seeds Hibiscus tiliaceus and its allied species: evidence from microsatellite analyses. Mol Ecol.

[50] Dodd RS, Afzal-Rafii Z, Kashani N, Budrick J (2002). Land barriers and open oceans: effects on gene diversity and population structure in *Avicennia germinans* L. (Avicenniaceae). Mol Ecol.

[51] Geng Q, Lian C, Goto S, Tao J, Kimura M, Islam MS (2008). Mating system, pollen and propagule dispersal, and spatial genetic structure in a high-density population of the mangrove tree *Kandelia candel*. Mol Ecol.

[52] Salas-Leiva D, Mayor-Durán V, Toro-Perea N (2009). Genetic diversity of black mangrove (*Avicennia germinans*) in natural and reforested areas of Salamanca Island Parkway. Colombian Caribbean Hydrobiologia.

[53] Van Oosterhout C, Weetman D, Hutchinson WF (2006). Estimation and adjustment of microsatellite null alleles in nonequilibrium populations. Mol Ecol Notes.

[54] Primack RB, Tomlinson PB (1980). Variation in Tropical Forest Breeding Systems. Biotropica.

[55] Ghosh A, Gupta S, Maity S, Das S (2008). Study of Floral Morphology of Some Indian Mangroves in Relation to Pollination. Res J Bot.

[56] Tyagi AP (2002). Cytogenetics and reproductive biology of mangroves in Rhizophoraceae. Aust J Bot.

[57] Coupland GT, Paling EI, McGuinness KA (2006). Floral abortion and pollination in four species of tropical mangroves from northern Australia. Aquat Bot.

[58] Duke N, Bunt J, Williams W (1984). Observations on the Floral and Vegetative Phenologies of North-Eastern Australian Mangroves. Aust J Bot.

[59] Bawa KS (1990). Plant-Pollinator Interactions in Tropical Rain Forests. Annu Rev Ecol Syst.

[60] Jump AS, Peñuelas J (2006). Genetic effects of chronic habitat fragmentation in a wind-pollinated tree. Proc Natl Acad Sci.

[61] Provan J, Beatty G, Hunter A, McDonald R, McLaughlin E, Preston SJ (2008). Restricted gene flow in fragmented populations of a wind-pollinated tree. Conserv Genet.

[62] Coyer J, Miller K, Engle J, Veldsink J, Cabello-Pasini A, Stam W (2008). Eelgrass meadows in the California Channel Islands and adjacent coast reveal a mosaic of two species, evidence for introgression and variable clonality. Ann Bot.

[63] Doyle JJ, Doyle JL (1987). A rapid DNA isolation procedure for small quantities of fresh leaf tissue. Phytochemical Bulletin.

[64] Shinmura Y, Wee A, Takayama K, Meenakshisundaram S, Asakawa T, Onrizal O (2012). Isolation and characterization of 14 microsatellite markers for *Rhizophora mucronata* (Rhizophoraceae) and their potential use in range-wide population studies. Conserv Genet Resour.

[65] Wee AKS, Takayama K, Kajita T, Webb EL (2013). Microsatellite loci for *Avicennia alba* (Acanthaceae), *Sonneratia alba* (Lythraceae) and *Rhizophora mucronata* (Rhizophoraceae). J Trop For Sci.

[66] Islam MS, Lian C, Kameyama N, Wu B, Hogetsu T (2004). Development of microsatellite markers in *Rhizophora stylosa* using a dual-suppression-polymerase chain reaction technique. Mol Ecol Notes.

[67] Takayama K, Tamura M, Tateishi Y, Kajita T (2009). Isolation and characterization of microsatellite loci in a mangrove species, *Rhizophora stylosa* (Rhizophoraceae). Conserv Genet Resour.

[68] Peakall R, Smouse P (2012). GenAlEx 6.5: Genetic analysis in Excel. Population genetic software for teaching and research – an update. Bioinformatics.

[69] FSTAT (Version 2.9.3.): A program to estimate and test gene diversities and fixation indices [http://www2.unil.ch/popgen/softwares/fstat.htm]

[70] Chapuis M-P, Estoup A (2007). Microsatellite Null Alleles and Estimation of Population Differentiation. Mol Biol Evol.

[71] Dempster AP, Laird NM, Rubin DB (1977). Maximum Likelihood from Incomplete Data via the EM Algorithm. J R Stat Soc Ser B Methodol.

[72] Raymond M, Rousset F (1995). GENEPOP (Version 1.2): Population genetics software for exact tests and ecumenicism. J Hered.

[73] Pritchard JK, Stephens M, Donnelly P (2000). Inference of Population Structure Using Multilocus Genotype Data. Genetics.

[74] Langella O (2002). POPULATIONS 1.2. 28. Population genetic software (individuals or populations distances, phylogenetic trees).

[75] Nei M, Tajima F, Tateno Y (1983). Accuracy of estimated phylogenetic trees from molecular data. J Mol Evol.

[76] Evanno G, Regnaut S, Goudet J (2005). Detecting the number of clusters of individuals using the software structure: a simulation study. Mol Ecol.

[77] Earl D, von Holdt B (2012). STRUCTURE HARVESTER: a website and program for visualizing STRUCTURE output and implementing the Evanno method. Conserv Genet Resour.

[78] Excoffier L, Lischer HEL (2010). Arlequin suite ver 3.5: a new series of programs to perform population genetics analyses under Linux and Windows. Mol Ecol Resour.

[79] Weir BS, Cockerham CC (1984). Estimating F-Statistics for the Analysis of Population Structure. Evolution.

[80] Wee AKS, Takayama K, Chua JL, Asakawa T, Meenakshisundaram SH, Onrizal, et. al, Webb EL Data from: Discerning two peas in a pod: Genetic differentiation and phylogeography of partially sympatric species complex Rhizophora mucronata Lam. and R. stylosa Griff. using SSR markers. Dryad Digital Repository. http://dx.doi.org/10.5061/dryad.42711 10.1186/s12862-015-0331-3 PMC4389924 25888261

