# Supplementary material for: Genetic differentiation and phylogeography of partially sympatric species complex Rhizophora mucronata Lam. and R. stylosa Griff. using SSR markers
Source: BMC Evol Biol. 2015 Mar 29;15:57. doi: 10.1186/s12862-015-0331-3 (PMC4389924; doi:10.1186/s12862-015-0331-3)
Supplement: Additional file 1: Table S1. — Genetic diversity parameters of the twenty microsatellite loci employed in this study. Table S2. Genetic diversity parameters of sampled populations. Table S3. Null allele frequency as estimated by FREENA for each population-locus comparison. Table S4. Pairwise FST estimates between all population pairs. [file 12862_2015_331_MOESM1_ESM.docx]

Table S1. Genetic diversity parameters of the twenty microsatellite loci employed in this study.

| Locus | N | N_RM_ | N_RS_ | Ar | Ho | He |
| --- | --- | --- | --- | --- | --- | --- |
| RM110 | 8 | 2 | 4 | 4.49 | 0.09 ± 0.03 | 0.14 ± 0.03 |
| RM102 | 13 | 4 | 3 | 5.84 | 0.09 ± 0.02 | 0.14 ± 0.03 |
| RM107 | 6 | 1 | 0 | 5.26 | 0.05 ± 0.01 | 0.08 ± 0.02 |
| RM103 | 8 | 1 | 1 | 5.81 | 0.08 ± 0.02 | 0.15 ± 0.04 |
| RM116 | 16 | 10 | 1 | 9.43 | 0.17 ± 0.03 | 0.33 ± 0.04 |
| RM114 | 10 | 4 | 3 | 4.66 | 0.10 ± 0.02 | 0.18 ± 0.03 |
| RM121 | 6 | 3 | 1 | 4.63 | 0.07 ± 0.02 | 0.12 ± 0.03 |
| RM111 | 8 | 3 | 0 | 5.22 | 0.08 ± 0.02 | 0.15 ± 0.03 |
| RM112 | 6 | 3 | 0 | 3.33 | 0.07 ± 0.02 | 0.10 ± 0.03 |
| RMu35 | 6 | 7 | 1 | 4.03 | 0.08 ± 0.02 | 0.12 ± 0.03 |
| RMu21 | 9 | 5 | 3 | 4.96 | 0.20 ± 0.03 | 0.34 ± 0.04 |
| RMu54 | 11 | 5 | 2 | 6.45 | 0.13 ± 0.04 | 0.23 ± 0.04 |
| Rhst11 | 14 | 5 | 3 | 7.17 | 0.17 ± 0.04 | 0.30 ± 0.04 |
| Rhst02 | 12 | 4 | 1 | 6.10 | 0.12 ± 0.03 | 0.21 ± 0.03 |
| Rhst15 | 10 | 3 | 0 | 6.13 | 0.05 ± 0.02 | 0.11 ± 0.03 |
| Rhst01 | 12 | 2 | 1 | 6.70 | 0.16 ± 0.03 | 0.25 ± 0.04 |
| Rhst13 | 15 | 6 | 1 | 7.94 | 0.09 ± 0.02 | 0.17 ± 0.03 |
| RS19 | 10 | 6 | 4 | 4.45 | 0.06 ± 0.02 | 0.14 ± 0.03 |
| RS59 | 11 | 5 | 2 | 6.19 | 0.15 ± 0.03 | 0.28 ± 0.04 |
| RS78 | 14 | 5 | 3 | 7.48 | 0.08 ± 0.02 | 0.23 ± 0.04 |
| Mean | 10.25 | 4.2 | 1.7 | 5.81 | 0.10 ± 0.01 | 0.19 ± 0.01 |

N, number of alleles; N_RM_; number of alleles unique to *R. mucronata*; N_RS_; number of alleles unique to *R. stylosa*; A_R_, allelic richness rarefied to a minimum of 15 diploid individuals; H*_O_*, observed heterozygosity; H*_E_*, expected heterozygosity.

.

Table S2. Genetic diversity parameters of sampled populations. Population codes are defined in Table 1.

| Pop. code |  | n | AR | Ho | He | FIS |
| --- | --- | --- | --- | --- | --- | --- |
| ***R. mucronata*** | | | | | | |
| MO1 |  | 34 | 1.92 | 0.06 ± 0.02 | 0.16 ± 0.05 | 0.62* |
| MO2 |  | 37 | 1.76 | 0.04 ± 0.01 | 0.13 ± 0.04 | 0.73* |
| MAU |  | 32 | 1.45 | 0.03 ± 0.01 | 0.07 ± 0.03 | 0.56* |
| SEY |  | 21 | 1.63 | 0.08 ± 0.03 | 0.18 ± 0.05 | 0.60* |
| ID1 |  | 32 | 1.86 | 0.07 ± 0.02 | 0.21 ± 0.05 | 0.68* |
| ID2 |  | 27 | 1.42 | 0.04 ± 0.02 | 0.10 ± 0.04 | 0.65* |
| MYA |  | 30 | 2.30 | 0.16 ± 0.04 | 0.24 ± 0.05 | 0.35* |
| IN1 |  | 33 | 1.14 | 0.02 ± 0.01 | 0.02 ± 0.01 | -0.05 |
| IN2 |  | 31 | 2.00 | 0.19 ± 0.03 | 0.25 ± 0.04 | 0.26* |
| IN3 |  | 27 | 1.42 | 0.03 ± 0.02 | 0.11 ± 0.04 | 0.72* |
| TH1 |  | 37 | 1.95 | 0.10 ± 0.03 | 0.21 ± 0.04 | 0.51* |
| TH2 |  | 31 | 1.86 | 0.09 ± 0.02 | 0.19 ± 0.04 | 0.54* |
| MA1 |  | 35 | 2.73 | 0.26 ± 0.03 | 0.34 ± 0.04 | 0.25* |
| MA2 |  | 39 | 2.32 | 0.20 ± 0.03 | 0.27 ± 0.04 | 0.27* |
| MA3 |  | 34 | 1.27 | 0.03 ± 0.02 | 0.05 ± 0.03 | 0.53* |
| MA4 |  | 33 | 1.55 | 0.09 ± 0.03 | 0.13 ± 0.04 | 0.34* |
| MA5 |  | 31 | 1.40 | 0.05 ± 0.02 | 0.11 ± 0.04 | 0.56* |
| TH3 |  | 32 | 1.58 | 0.05 ± 0.02 | 0.11 ± 0.04 | 0.54* |
| VI1 |  | 38 | 1.15 | 0.01 ± 0.01 | 0.05 ± 0.03 | 0.89* |
| MA6 |  | 30 | 2.14 | 0.20 ± 0.04 | 0.27 ± 0.04 | 0.29* |
| PH1 |  | 30 | 2.23 | 0.16 ± 0.03 | 0.25 ± 0.05 | 0.38* |
| IN4 |  | 35 | 3.03 | 0.10 ± 0.02 | 0.49 ± 0.04 | 0.80* |
| PA1 |  | 34 | 2.29 | 0.45 ± 0.09 | 0.34 ± 0.06 | -0.32 |
| PA2 |  | 23 | 1.45 | 0.10 ± 0.03 | 0.13 ± 0.04 | 0.29 |
|  | Mean | 31.9 | 1.83 | 0.11 ± 0.01 | 0.18 ± 0.01 |  |
|  |  |  |  |  |  |  |
| ***R. stylosa*** | | | | | | |
| IN2 |  | 22 | 1.58 | 0.03 ± 0.01 | 0.14 ± 0.05 | 0.83* |
| VI2 |  | 47 | 1.44 | 0.03 ± 0.01 | 0.11 ± 0.04 | 0.74* |
| PH2 |  | 32 | 1.57 | 0.07 ± 0.02 | 0.15 ± 0.05 | 0.55* |
| JA1 |  | 30 | 1.54 | 0.03 ± 0.01 | 0.12 ± 0.04 | 0.72* |
| JA2 |  | 29 | 1.44 | 0.05 ± 0.01 | 0.13 ± 0.04 | 0.66* |
| JA3 |  | 30 | 1.06 | 0.01 ± 0.00 | 0.01 ± 0.00 | -0.01 |
| MIC |  | 31 | 2.25 | 0.13 ± 0.01 | 0.44 ± 0.04 | 0.72* |
| NCL |  | 28 | 1.60 | 0.11 ± 0.03 | 0.15 ± 0.04 | 0.25 |
| VA1 |  | 30 | 1.72 | 0.17 ± 0.05 | 0.19 ± 0.05 | 0.11 |
| VA2 |  | 19 | 1.49 | 0.12 ± 0.04 | 0.15 ± 0.05 | 0.21 |
| FI1 |  | 16 | 2.39 | 0.29 ± 0.04 | 0.38 ± 0.04 | 0.26* |
| FI2 |  | 27 | 2.23 | 0.13 ± 0.02 | 0.37 ± 0.04 | 0.66* |
|  | Mean | 28.4 | 1.69 | 0.10 ± 0.01 | 0.19 ± 0.01 |  |

*n*, number of samples; A*_R_*, allelic richness rarefied to a minimum of 15 diploid individuals; H*_O_*, observed heterozygosity; H*_E_*, expected heterozygosity; F*_IS_*, inbreeding coefficient; *significant at

P < 0.05.

Table S3. Null allele frequency as estimated by FREENA for each population-locus comparison.

|  | **MO1** | **MO2** | **MAU** | **SEY** | **ID1** | **ID2** | **MYA** | **IN1** | **IN2** | **IN3** | **TH1** | **TH2** | **MA1** | **MA2** | **MA3** | **MA4** | **MA5** | **TH3** | **VI1** | **MA6** | **PH1** | **IN4** | **PA1** | **PA2** | **IN2** | **VI2** | **PH2** | **JA1** | **JA2** | **JA3** | **MIC** | **NCL** | **VA1** | **VA2** | **FI1** | **FI2** |
| --- | --- | --- | --- | --- | --- | --- | --- | --- | --- | --- | --- | --- | --- | --- | --- | --- | --- | --- | --- | --- | --- | --- | --- | --- | --- | --- | --- | --- | --- | --- | --- | --- | --- | --- | --- | --- |
| RM110 | 0.00 | 0.00 | 0.00 | 0.00 | 0.00 | 0.00 | 0.18 | 0.00 | 0.07 | 0.00 | 0.00 | 0.00 | 0.00 | 0.00 | 0.00 | 0.00 | 0.00 | 0.00 | 0.00 | 0.13 | 0.11 | 0.00 | 0.00 | 0.00 | 0.00 | 0.20 | 0.00 | 0.00 | 0.13 | 0.00 | 0.20 | 0.07 | 0.00 | 0.00 | 0.10 | 0.28 |
| RM102 | 0.00 | 0.10 | 0.00 | 0.00 | 0.00 | 0.00 | 0.10 | 0.00 | 0.07 | 0.00 | 0.15 | 0.11 | 0.06 | 0.00 | 0.00 | 0.00 | 0.00 | 0.11 | 0.00 | 0.17 | 0.00 | 0.25 | 0.00 | 0.00 | 0.00 | 0.00 | 0.13 | 0.00 | 0.00 | 0.00 | 0.23 | 0.00 | 0.02 | 0.13 | 0.08 | 0.13 |
| RM107 | 0.00 | 0.00 | 0.00 | 0.00 | 0.00 | 0.00 | 0.00 | 0.00 | 0.05 | 0.00 | 0.07 | 0.00 | 0.00 | 0.00 | 0.00 | 0.00 | 0.00 | 0.00 | 0.00 | 0.00 | 0.00 | 0.25 | 0.00 | 0.00 | 0.14 | 0.00 | 0.13 | 0.05 | 0.17 | 0.00 | 0.25 | 0.00 | 0.00 | 0.00 | 0.00 | 0.00 |
| RM103 | 0.00 | 0.00 | 0.00 | 0.00 | 0.00 | 0.00 | 0.00 | 0.00 | 0.04 | 0.00 | 0.08 | 0.00 | 0.07 | 0.00 | 0.00 | 0.00 | 0.00 | 0.00 | 0.00 | 0.11 | 0.00 | 0.32 | 0.25 | 0.07 | 0.00 | 0.00 | 0.28 | 0.00 | 0.00 | 0.00 | 0.27 | 0.00 | 0.12 | 0.00 | 0.00 | 0.22 |
| RM116 | 0.22 | 0.29 | 0.00 | 0.23 | 0.30 | 0.20 | 0.17 | 0.00 | 0.00 | 0.25 | 0.20 | 0.26 | 0.06 | 0.05 | 0.10 | 0.02 | 0.23 | 0.19 | 0.00 | 0.09 | 0.00 | 0.30 | 0.00 | 0.12 | 0.00 | 0.10 | 0.00 | 0.00 | 0.26 | 0.00 | 0.23 | 0.17 | 0.00 | 0.00 | 0.00 | 0.25 |
| RM114 | 0.00 | 0.00 | 0.00 | 0.27 | 0.13 | 0.00 | 0.12 | 0.00 | 0.01 | 0.00 | 0.00 | 0.00 | 0.15 | 0.18 | 0.12 | 0.16 | 0.05 | 0.11 | 0.00 | 0.20 | 0.00 | 0.26 | 0.00 | 0.00 | 0.00 | 0.00 | 0.00 | 0.00 | 0.00 | 0.00 | 0.32 | 0.03 | 0.00 | 0.00 | 0.26 | 0.10 |
| RM121 | 0.00 | 0.00 | 0.00 | 0.00 | 0.00 | 0.00 | 0.00 | 0.00 | 0.01 | 0.02 | 0.12 | 0.00 | 0.11 | 0.12 | 0.00 | 0.00 | 0.00 | 0.00 | 0.00 | 0.00 | 0.21 | 0.27 | 0.00 | 0.00 | 0.00 | 0.00 | 0.00 | 0.00 | 0.00 | 0.00 | 0.09 | 0.00 | 0.13 | 0.00 | 0.12 | 0.19 |
| RM111 | 0.00 | 0.00 | 0.00 | 0.00 | 0.08 | 0.11 | 0.07 | 0.00 | 0.00 | 0.20 | 0.25 | 0.16 | 0.09 | 0.04 | 0.00 | 0.00 | 0.00 | 0.00 | 0.00 | 0.00 | 0.13 | 0.24 | 0.00 | 0.12 | 0.00 | 0.00 | 0.00 | 0.12 | 0.00 | 0.00 | 0.31 | 0.00 | 0.00 | 0.00 | 0.00 | 0.11 |
| RM112 | 0.21 | 0.13 | 0.00 | 0.14 | 0.00 | 0.00 | 0.00 | 0.00 | 0.14 | 0.00 | 0.00 | 0.00 | 0.10 | 0.07 | 0.00 | 0.00 | 0.00 | 0.00 | 0.00 | 0.00 | 0.00 | 0.25 | 0.00 | 0.00 | 0.00 | 0.00 | 0.00 | 0.00 | 0.00 | 0.00 | 0.17 | 0.00 | 0.00 | 0.00 | 0.00 | 0.16 |
| RMu35 | 0.14 | 0.26 | 0.12 | 0.22 | 0.15 | 0.00 | 0.11 | 0.00 | 0.14 | 0.31 | 0.00 | 0.17 | 0.09 | 0.05 | 0.00 | 0.14 | 0.10 | 0.11 | 0.31 | 0.01 | 0.00 | 0.30 | 0.00 | 0.11 | 0.00 | 0.00 | 0.18 | 0.23 | 0.19 | 0.00 | 0.24 | 0.00 | 0.00 | 0.00 | 0.01 | 0.18 |
| RMu21 | 0.12 | 0.11 | 0.00 | 0.13 | 0.27 | 0.15 | 0.17 | 0.00 | 0.00 | 0.00 | 0.13 | 0.00 | 0.00 | 0.00 | 0.00 | 0.00 | 0.00 | 0.00 | 0.00 | 0.10 | 0.03 | 0.25 | 0.00 | 0.00 | 0.29 | 0.21 | 0.11 | 0.00 | 0.00 | 0.00 | 0.25 | 0.00 | 0.03 | 0.13 | 0.13 | 0.28 |
| RMu54 | 0.00 | 0.00 | 0.00 | 0.00 | 0.25 | 0.00 | 0.13 | 0.00 | 0.00 | 0.00 | 0.13 | 0.15 | 0.05 | 0.00 | 0.00 | 0.00 | 0.00 | 0.07 | 0.00 | 0.00 | 0.00 | 0.24 | 0.02 | 0.00 | 0.36 | 0.26 | 0.00 | 0.20 | 0.00 | 0.00 | 0.00 | 0.00 | 0.00 | 0.00 | 0.00 | 0.00 |
| Rhst11 | 0.26 | 0.19 | 0.00 | 0.00 | 0.20 | 0.11 | 0.07 | 0.00 | 0.00 | 0.00 | 0.06 | 0.13 | 0.00 | 0.02 | 0.00 | 0.00 | 0.00 | 0.17 | 0.00 | 0.04 | 0.28 | 0.36 | 0.00 | 0.00 | 0.00 | 0.00 | 0.10 | 0.17 | 0.22 | 0.00 | 0.19 | 0.00 | 0.09 | 0.02 | 0.01 | 0.00 |
| Rhst02 | 0.00 | 0.00 | 0.21 | 0.16 | 0.00 | 0.00 | 0.00 | 0.00 | 0.00 | 0.00 | 0.15 | 0.00 | 0.11 | 0.08 | 0.00 | 0.00 | 0.00 | 0.00 | 0.00 | 0.11 | 0.00 | 0.27 | 0.00 | 0.00 | 0.00 | 0.10 | 0.00 | 0.22 | 0.22 | 0.00 | 0.29 | 0.08 | 0.00 | 0.00 | 0.14 | 0.27 |
| Rhst15 | 0.00 | 0.00 | 0.00 | 0.00 | 0.00 | 0.00 | 0.15 | 0.00 | 0.12 | 0.00 | 0.00 | 0.00 | 0.10 | 0.07 | 0.00 | 0.00 | 0.00 | 0.00 | 0.00 | 0.05 | 0.00 | 0.21 | 0.00 | 0.00 | 0.00 | 0.00 | 0.00 | 0.00 | 0.00 | 0.00 | 0.20 | 0.00 | 0.00 | 0.00 | 0.12 | 0.15 |
| Rhst01 | 0.16 | 0.14 | 0.12 | 0.18 | 0.25 | 0.20 | 0.00 | 0.00 | 0.17 | 0.00 | 0.20 | 0.13 | 0.07 | 0.04 | 0.13 | 0.10 | 0.21 | 0.25 | 0.00 | 0.09 | 0.17 | 0.26 | 0.00 | 0.00 | 0.27 | 0.26 | 0.00 | 0.00 | 0.00 | 0.00 | 0.17 | 0.11 | 0.00 | 0.11 | 0.03 | 0.22 |
| Rhst13 | 0.20 | 0.19 | 0.09 | 0.20 | 0.00 | 0.30 | 0.16 | 0.00 | 0.00 | 0.00 | 0.14 | 0.00 | 0.03 | 0.06 | 0.00 | 0.00 | 0.00 | 0.00 | 0.00 | 0.00 | 0.08 | 0.34 | 0.00 | 0.08 | 0.33 | 0.20 | 0.00 | 0.00 | 0.00 | 0.00 | 0.27 | 0.00 | 0.00 | 0.12 | 0.10 | 0.27 |
| RS19 | 0.10 | 0.00 | 0.15 | 0.00 | 0.12 | 0.00 | 0.00 | 0.00 | 0.08 | 0.10 | 0.17 | 0.16 | 0.07 | 0.06 | 0.00 | 0.14 | 0.00 | 0.00 | 0.00 | 0.11 | 0.00 | 0.28 | 0.00 | 0.10 | 0.00 | 0.00 | 0.19 | 0.26 | 0.15 | 0.00 | 0.23 | 0.00 | 0.00 | 0.00 | 0.00 | 0.07 |
| RS59 | 0.21 | 0.25 | 0.00 | 0.00 | 0.27 | 0.00 | 0.10 | 0.00 | 0.14 | 0.33 | 0.11 | 0.23 | 0.14 | 0.14 | 0.21 | 0.11 | 0.22 | 0.08 | 0.30 | 0.09 | 0.00 | 0.31 | 0.00 | 0.00 | 0.00 | 0.00 | 0.00 | 0.00 | 0.00 | 0.00 | 0.19 | 0.06 | 0.00 | 0.00 | 0.00 | 0.24 |
| RS78 | 0.00 | 0.08 | 0.14 | 0.21 | 0.22 | 0.00 | 0.00 | 0.00 | 0.00 | 0.00 | 0.00 | 0.20 | 0.11 | 0.19 | 0.00 | 0.07 | 0.17 | 0.00 | 0.00 | 0.14 | 0.33 | 0.34 | 0.00 | 0.00 | 0.28 | 0.12 | 0.11 | 0.21 | 0.08 | 0.00 | 0.30 | 0.00 | 0.10 | 0.15 | 0.33 | 0.33 |

Table S4. Pairwise F_ST_ estimates between all population pairs are listed below the diagonal. Significance values (after Bonferroni correction) are listed above the diagonal. Population codes are defined in Table 1. Warmer colours represent lower F_ST_ values; cooler colours represent higher F_ST_ values.

|  |  | **MO1** | **MO2** | **MAU** | **SEY** | **ID1** | **ID2** | **MYA** | **IN1** | **IN2** | **IN3** | **TH1** | **TH2** | **MA1** | **MA2** | **MA3** | **MA4** | **MA5** | **TH3** | **VI1** | **MA6** | **PH1** | **IN4** | **PA1** | **PA2** | **IN2** | **VI2** | **PH2** | **JA1** | **JA2** | **JA3** | **MIC** | **NCL** | **VA1** | **VA2** | **FI1** | **FI2** |
| --- | --- | --- | --- | --- | --- | --- | --- | --- | --- | --- | --- | --- | --- | --- | --- | --- | --- | --- | --- | --- | --- | --- | --- | --- | --- | --- | --- | --- | --- | --- | --- | --- | --- | --- | --- | --- | --- |
| **1** | **MO1** |  | *** | *** | *** | *** | *** | *** | *** | *** | *** | *** | *** | *** | *** | *** | *** | *** | *** | *** | *** | *** | *** | *** | *** | *** | *** | *** | *** | *** | *** | *** | *** | *** | *** | *** | *** |
| **2** | **MO2** | 0.35 |  | *** | *** | *** | *** | *** | *** | *** | *** | *** | *** | *** | *** | *** | *** | *** | *** | *** | *** | *** | *** | *** | *** | *** | *** | *** | *** | *** | *** | *** | *** | *** | *** | *** | *** |
| **3** | **MAU** | 0.57 | 0.54 |  | *** | *** | *** | *** | *** | *** | *** | *** | *** | *** | *** | *** | *** | *** | *** | *** | *** | *** | *** | *** | *** | *** | *** | *** | *** | *** | *** | *** | *** | *** | *** | *** | *** |
| **4** | **SEY** | 0.45 | 0.52 | 0.57 |  | *** | *** | *** | *** | *** | *** | *** | *** | *** | *** | *** | *** | *** | *** | *** | *** | *** | *** | *** | *** | *** | *** | *** | *** | *** | *** | *** | *** | *** | *** | *** | *** |
| **5** | **ID1** | 0.68 | 0.70 | 0.75 | 0.69 |  | *** | *** | *** | *** | *** | *** | *** | *** | *** | *** | *** | *** | *** | *** | *** | *** | *** | *** | *** | *** | *** | *** | *** | *** | *** | *** | *** | *** | *** | *** | *** |
| **6** | **ID2** | 0.77 | 0.77 | 0.84 | 0.78 | 0.61 |  | *** | *** | *** | *** | *** | *** | *** | *** | *** | *** | *** | *** | *** | *** | *** | *** | *** | *** | *** | *** | *** | *** | *** | *** | *** | *** | *** | *** | *** | *** |
| **7** | **MYA** | 0.71 | 0.74 | 0.78 | 0.69 | 0.63 | 0.64 |  | *** | *** | *** | *** | *** | *** | *** | *** | *** | *** | *** | *** | *** | *** | *** | *** | *** | *** | *** | *** | *** | *** | *** | *** | *** | *** | *** | *** | *** |
| **8** | **IN1** | 0.86 | 0.90 | 0.94 | 0.88 | 0.82 | 0.91 | 0.65 |  | *** | *** | *** | *** | *** | *** | *** | *** | *** | *** | *** | *** | *** | *** | *** | *** | *** | *** | *** | *** | *** | *** | *** | *** | *** | *** | *** | *** |
| **9** | **IN2** | 0.70 | 0.75 | 0.77 | 0.69 | 0.65 | 0.72 | 0.41 | 0.28 |  | *** | *** | *** | *** | *** | *** | *** | *** | *** | *** | *** | *** | *** | *** | *** | *** | *** | *** | *** | *** | *** | *** | *** | *** | *** | *** | *** |
| **10** | **IN3** | 0.80 | 0.82 | 0.87 | 0.81 | 0.74 | 0.84 | 0.72 | 0.86 | 0.63 |  | *** | *** | *** | *** | *** | *** | *** | *** | *** | *** | *** | *** | *** | *** | *** | *** | *** | *** | *** | *** | *** | *** | *** | *** | *** | *** |
| **11** | **TH1** | 0.75 | 0.78 | 0.81 | 0.76 | 0.68 | 0.77 | 0.63 | 0.76 | 0.59 | 0.34 |  | *** | *** | *** | *** | *** | *** | *** | *** | *** | *** | *** | *** | *** | *** | *** | *** | *** | *** | *** | *** | *** | *** | *** | *** | *** |
| **12** | **TH2** | 0.77 | 0.79 | 0.83 | 0.77 | 0.71 | 0.80 | 0.69 | 0.82 | 0.63 | 0.31 | 0.10 |  | *** | *** | *** | *** | *** | *** | *** | *** | *** | *** | *** | *** | *** | *** | *** | *** | *** | *** | *** | *** | *** | *** | *** | *** |
| **13** | **MA1** | 0.67 | 0.70 | 0.72 | 0.64 | 0.64 | 0.71 | 0.60 | 0.69 | 0.51 | 0.31 | 0.27 | 0.17 |  | NS | *** | *** | *** | *** | *** | *** | *** | *** | *** | *** | *** | *** | *** | *** | *** | *** | *** | *** | *** | *** | *** | *** |
| **14** | **MA2** | 0.71 | 0.74 | 0.76 | 0.69 | 0.69 | 0.76 | 0.66 | 0.76 | 0.59 | 0.38 | 0.35 | 0.24 | 0.01 |  | *** | *** | *** | *** | *** | *** | *** | *** | *** | *** | *** | *** | *** | *** | *** | *** | *** | *** | *** | *** | *** | *** |
| **15** | **MA3** | 0.87 | 0.88 | 0.92 | 0.88 | 0.80 | 0.89 | 0.79 | 0.94 | 0.75 | 0.58 | 0.31 | 0.23 | 0.38 | 0.44 |  | *** | *** | *** | *** | *** | *** | *** | *** | *** | *** | *** | *** | *** | *** | *** | *** | *** | *** | *** | *** | *** |
| **16** | **MA4** | 0.81 | 0.83 | 0.87 | 0.82 | 0.74 | 0.84 | 0.73 | 0.87 | 0.68 | 0.41 | 0.14 | 0.08 | 0.26 | 0.31 | 0.23 |  | *** | *** | *** | *** | *** | *** | *** | *** | *** | *** | *** | *** | *** | *** | *** | *** | *** | *** | *** | *** |
| **17** | **MA5** | 0.83 | 0.85 | 0.88 | 0.83 | 0.76 | 0.86 | 0.76 | 0.89 | 0.69 | 0.48 | 0.32 | 0.19 | 0.27 | 0.31 | 0.43 | 0.25 |  | *** | *** | *** | *** | *** | *** | *** | *** | *** | *** | *** | *** | *** | *** | *** | *** | *** | *** | *** |
| **18** | **TH3** | 0.83 | 0.85 | 0.89 | 0.84 | 0.76 | 0.86 | 0.75 | 0.89 | 0.70 | 0.45 | 0.20 | 0.12 | 0.29 | 0.34 | 0.23 | 0.12 | 0.21 |  | *** | *** | *** | *** | *** | *** | *** | *** | *** | *** | *** | *** | *** | *** | *** | *** | *** | *** |
| **19** | **VI1** | 0.87 | 0.88 | 0.92 | 0.88 | 0.82 | 0.91 | 0.81 | 0.94 | 0.76 | 0.46 | 0.44 | 0.28 | 0.32 | 0.37 | 0.56 | 0.42 | 0.46 | 0.40 |  | *** | *** | *** | *** | *** | *** | *** | *** | *** | *** | *** | *** | *** | *** | *** | *** | *** |
| **20** | **MA6** | 0.73 | 0.76 | 0.79 | 0.72 | 0.67 | 0.76 | 0.65 | 0.78 | 0.60 | 0.44 | 0.26 | 0.23 | 0.22 | 0.27 | 0.37 | 0.18 | 0.34 | 0.34 | 0.48 |  | *** | *** | *** | *** | *** | *** | *** | *** | *** | *** | *** | *** | *** | *** | *** | *** |
| **21** | **PH1** | 0.58 | 0.61 | 0.63 | 0.54 | 0.53 | 0.60 | 0.53 | 0.65 | 0.48 | 0.47 | 0.42 | 0.41 | 0.31 | 0.35 | 0.50 | 0.43 | 0.48 | 0.48 | 0.53 | 0.27 |  | *** | *** | *** | *** | *** | *** | *** | *** | *** | *** | *** | *** | *** | *** | *** |
| **22** | **IN4** | 0.75 | 0.79 | 0.81 | 0.73 | 0.73 | 0.79 | 0.68 | 0.81 | 0.64 | 0.70 | 0.64 | 0.64 | 0.48 | 0.51 | 0.76 | 0.69 | 0.70 | 0.70 | 0.77 | 0.60 | 0.51 |  | *** | *** | *** | *** | *** | *** | *** | *** | *** | *** | *** | *** | *** | *** |
| **23** | **PA1** | 0.69 | 0.71 | 0.75 | 0.67 | 0.63 | 0.70 | 0.67 | 0.79 | 0.64 | 0.70 | 0.67 | 0.67 | 0.58 | 0.62 | 0.76 | 0.71 | 0.72 | 0.72 | 0.76 | 0.62 | 0.44 | 0.67 |  | *** | *** | *** | *** | *** | *** | *** | *** | *** | *** | *** | *** | *** |
| **24** | **PA2** | 0.80 | 0.82 | 0.87 | 0.80 | 0.76 | 0.85 | 0.77 | 0.91 | 0.71 | 0.68 | 0.63 | 0.59 | 0.43 | 0.47 | 0.76 | 0.66 | 0.68 | 0.70 | 0.72 | 0.51 | 0.33 | 0.71 | 0.66 |  | *** | *** | *** | *** | *** | *** | *** | *** | *** | *** | *** | *** |
| **25** | **IN2** | 0.83 | 0.85 | 0.88 | 0.81 | 0.80 | 0.87 | 0.79 | 0.92 | 0.76 | 0.87 | 0.81 | 0.82 | 0.73 | 0.76 | 0.91 | 0.86 | 0.87 | 0.87 | 0.91 | 0.77 | 0.57 | 0.78 | 0.63 | 0.85 |  | *** | *** | *** | *** | *** | *** | *** | *** | *** | *** | *** |
| **26** | **VI2** | 0.85 | 0.87 | 0.89 | 0.84 | 0.83 | 0.89 | 0.83 | 0.92 | 0.81 | 0.88 | 0.84 | 0.85 | 0.78 | 0.80 | 0.91 | 0.88 | 0.88 | 0.89 | 0.91 | 0.82 | 0.65 | 0.82 | 0.68 | 0.87 | 0.47 |  | *** | *** | *** | *** | *** | *** | *** | *** | *** | *** |
| **27** | **PH2** | 0.83 | 0.85 | 0.87 | 0.80 | 0.80 | 0.86 | 0.80 | 0.91 | 0.78 | 0.87 | 0.82 | 0.83 | 0.74 | 0.77 | 0.90 | 0.86 | 0.87 | 0.87 | 0.90 | 0.78 | 0.59 | 0.78 | 0.64 | 0.85 | 0.49 | 0.61 |  | *** | *** | *** | *** | *** | *** | *** | *** | *** |
| **28** | **JA1** | 0.83 | 0.85 | 0.89 | 0.82 | 0.81 | 0.88 | 0.81 | 0.93 | 0.78 | 0.87 | 0.82 | 0.83 | 0.74 | 0.77 | 0.91 | 0.86 | 0.87 | 0.88 | 0.91 | 0.78 | 0.57 | 0.80 | 0.61 | 0.85 | 0.68 | 0.69 | 0.66 |  | *** | *** | *** | *** | *** | *** | *** | *** |
| **29** | **JA2** | 0.83 | 0.85 | 0.88 | 0.81 | 0.81 | 0.87 | 0.80 | 0.92 | 0.78 | 0.87 | 0.82 | 0.83 | 0.74 | 0.77 | 0.91 | 0.86 | 0.87 | 0.88 | 0.91 | 0.78 | 0.59 | 0.79 | 0.63 | 0.85 | 0.67 | 0.69 | 0.63 | 0.43 |  | *** | *** | *** | *** | *** | *** | *** |
| **30** | **JA3** | 0.90 | 0.91 | 0.95 | 0.90 | 0.86 | 0.94 | 0.86 | 0.99 | 0.85 | 0.94 | 0.87 | 0.89 | 0.80 | 0.83 | 0.97 | 0.92 | 0.93 | 0.94 | 0.97 | 0.85 | 0.65 | 0.87 | 0.73 | 0.93 | 0.81 | 0.80 | 0.81 | 0.78 | 0.75 |  | *** | *** | *** | *** | *** | *** |
| **31** | **MIC** | 0.61 | 0.65 | 0.68 | 0.57 | 0.62 | 0.67 | 0.58 | 0.69 | 0.53 | 0.60 | 0.56 | 0.56 | 0.46 | 0.50 | 0.65 | 0.61 | 0.62 | 0.62 | 0.67 | 0.50 | 0.35 | 0.52 | 0.45 | 0.60 | 0.60 | 0.67 | 0.60 | 0.61 | 0.62 | 0.70 |  | *** | *** | *** | *** | *** |
| **32** | **NCL** | 0.81 | 0.83 | 0.87 | 0.80 | 0.78 | 0.85 | 0.78 | 0.91 | 0.77 | 0.83 | 0.78 | 0.79 | 0.69 | 0.73 | 0.88 | 0.82 | 0.84 | 0.84 | 0.88 | 0.74 | 0.59 | 0.77 | 0.67 | 0.83 | 0.83 | 0.86 | 0.83 | 0.82 | 0.82 | 0.91 | 0.62 |  | *** | *** | *** | *** |
| **33** | **VA1** | 0.80 | 0.82 | 0.84 | 0.77 | 0.74 | 0.82 | 0.75 | 0.89 | 0.74 | 0.80 | 0.75 | 0.76 | 0.66 | 0.70 | 0.85 | 0.79 | 0.80 | 0.81 | 0.85 | 0.71 | 0.56 | 0.74 | 0.63 | 0.80 | 0.77 | 0.81 | 0.77 | 0.80 | 0.78 | 0.86 | 0.58 | 0.70 |  | NS | *** | *** |
| **34** | **VA2** | 0.82 | 0.84 | 0.87 | 0.79 | 0.76 | 0.85 | 0.77 | 0.92 | 0.75 | 0.83 | 0.77 | 0.78 | 0.67 | 0.71 | 0.89 | 0.82 | 0.83 | 0.84 | 0.89 | 0.72 | 0.55 | 0.76 | 0.64 | 0.83 | 0.80 | 0.83 | 0.80 | 0.83 | 0.81 | 0.91 | 0.58 | 0.74 | 0.04 |  | *** | *** |
| **35** | **FI1** | 0.73 | 0.75 | 0.78 | 0.68 | 0.66 | 0.75 | 0.67 | 0.84 | 0.66 | 0.73 | 0.68 | 0.68 | 0.58 | 0.62 | 0.79 | 0.72 | 0.73 | 0.74 | 0.80 | 0.62 | 0.45 | 0.66 | 0.54 | 0.72 | 0.70 | 0.77 | 0.71 | 0.73 | 0.71 | 0.83 | 0.47 | 0.48 | 0.32 | 0.35 |  | *** |
| **36** | **FI2** | 0.71 | 0.73 | 0.76 | 0.67 | 0.64 | 0.71 | 0.66 | 0.81 | 0.65 | 0.70 | 0.66 | 0.66 | 0.58 | 0.61 | 0.75 | 0.70 | 0.70 | 0.71 | 0.77 | 0.61 | 0.47 | 0.65 | 0.53 | 0.70 | 0.67 | 0.73 | 0.68 | 0.70 | 0.68 | 0.76 | 0.46 | 0.57 | 0.24 | 0.26 | 0.16 |  |

NS, not significant; ***significant at P < 0.001
